# Milk Bottom-Up Proteomics: Method Optimization

**DOI:** 10.3389/fgene.2015.00360

**Published:** 2016-01-11

**Authors:** Delphine Vincent, Vilnis Ezernieks, Aaron Elkins, Nga Nguyen, Peter J. Moate, Benjamin G. Cocks, Simone Rochfort

**Affiliations:** ^1^Department of Economic Development, Jobs, Transport and Resources, AgriBio Centre, La Trobe UniversityBundoora, VIC, Australia; ^2^Department of Economic Development, Jobs, Transport and ResourcesEllinbank, VIC, Australia; ^3^School of Applied Systems Biology, La Trobe UniversityBundoora, VIC, Australia

**Keywords:** Jersey and Holstein-Friesian cow milk, shotgun nLC-ESI-MS, proteome, trypsin digestion, replicates

## Abstract

Milk is a complex fluid whose proteome displays a diverse set of proteins of high abundance such as caseins and medium to low abundance whey proteins such as ß-lactoglobulin, lactoferrin, immunoglobulins, glycoproteins, peptide hormones, and enzymes. A sample preparation method that enables high reproducibility and throughput is key in reliably identifying proteins present or proteins responding to conditions such as a diet, health or genetics. Using skim milk samples from Jersey and Holstein-Friesian cows, we compared three extraction procedures which have not previously been applied to samples of cows' milk. Method A (urea) involved a simple dilution of the milk in a urea-based buffer, method B (TCA/acetone) involved a trichloroacetic acid (TCA)/acetone precipitation, and method C (methanol/chloroform) involved a tri-phasic partition method in chloroform/methanol solution. Protein assays, SDS-PAGE profiling, and trypsin digestion followed by nanoHPLC-electrospray ionization-tandem mass spectrometry (nLC-ESI-MS/MS) analyses were performed to assess their efficiency. Replicates were used at each analytical step (extraction, digestion, injection) to assess reproducibility. Mass spectrometry (MS) data are available via ProteomeXchange with identifier PXD002529. Overall 186 unique accessions, major and minor proteins, were identified with a combination of methods. Method C (methanol/chloroform) yielded the best resolved SDS-patterns and highest protein recovery rates, method A (urea) yielded the greatest number of accessions, and, of the three procedures, method B (TCA/acetone) was the least compatible of all with a wide range of downstream analytical procedures. Our results also highlighted breed differences between the proteins in milk of Jersey and Holstein-Friesian cows.

## Introduction

Milk is a very complex body fluid whose primary biological function is to nurture newborns. Cow's milk, in its pure form or derivative dairy products such as cream, butter, cheese, and yogurt, is a major source of nutrition for humans. On average, cow's milk is composed of 88% of water, 4.8% carbohydrates, 3.9% lipids, 3.2% proteins, and 0.7% minerals (Jost, 2005). *Bos taurus* have been bred for millenia and selected to increase milk production in dairy animals.

The recent sequencing of *Bos taurus* genome (Bovine Genome Sequencing and Analysis Consortium, 2009) paved the way for omics studies, particularly proteomics which heavily relies on gene model annotations for accurate protein identification. The cattle genome is predicted to contain at least 22,000 protein-coding genes. In cow's milk, the most abundant proteins are caseins (α-S1-, α-S2-, β-, and κ-forms) which represent about 78% of total protein concentration, followed by whey proteins which make up 17% (β-lactoglobulin, α-lactalbumin, lactoferrin, and lactoperoxidase) (reviewed in Bendixen et al., 2011; Roncada et al., 2012).

Various protocols for milk protein extraction have been described in the literature including dilution of skim milk in a urea-based buffer compatible with isoelectric focusing (IEF; Boehmer et al., 2008; Jensen et al., 2012a), acetone precipitation of full cream milk (Danielsen et al., 2010), ultracentrifugation to pellet caseins (Hettinga et al., 2011; Kim et al., 2011; Reinhardt et al., 2013) followed by 10 kD molecular weight cut-off (MWCO) filtration of whey fraction (Le et al., 2011), ammonium sulfate precipitation of caseins to isolate serum (Hogarth et al., 2004), acetic acid removal of caseins to isolate whey proteins (Senda et al., 2011), or low speed centrifugation to remove the fat layer followed by a dilution of the skim milk in a protein buffer compatible with 2-DE (Yang et al., 2013). The diversity of methods led us to assume there was not one established method proven to be superior to the others for enabling a complete proteome analysis while ensuring high throughput. Recently, Nissen et al. (2012, 2013) applied a fractionation method to bovine colostrum or mature milk resulting in a cell-free and fat-free fraction, a cell pellet fraction, and a whey fraction which was further treated by acidification, ultrafiltration or centrifugation. In these studies, the proteins from the various fractions were trypsin-digested, analyzed using 2-D-LC-MS/MS, and compared to the corresponding non-fractionated milk proteome. With this strategy, the authors deepened milk proteome coverage by identifying 69 (17%) additional proteins in the fractionated samples compared to the non-fractionated ones where 334 proteins could be identified (Nissen et al., 2012). However this coverage was achieved at the expense of throughput. We are currently undertaking a vast systems biology project aiming at characterizing milk from two widely-studied bovine breeds: Holstein-Friesian and Jersey. The first step was to optimize the extraction method for the proteomics aspect of the project. Because our literature survey failed to find publications describing attempts to optimize protein extraction from cow milk by comparing several protocols, compounded by the fact that there was no consensus on which protein extraction method to use to analyse the cow milk proteome, we designed an experiment to compare different extraction procedures used to recover as many proteins as possible for their analysis by shotgun LC-MS/MS in a high throughput fashion.

To this end, we used three very different methods that have not been used in a gel-free bottom-up approach before to extract proteins from cow's skim milk from two different breeds. Replicates were used during the extraction, digestion, as well as injection steps to assess the reproducibility of the methods. Our null hypothesis was that the three methods would be similar in their major attributes when used to analyse proteins in milk samples from Jersey and Friesian-Holstein cows. These attributes include method efficiency as measured by the concentration of extracted protein, the SDS-PAGE patterns, the number of protein accessions identified following trypsin digestion and nLC-ESI-MS/MS analyses, cost of the extraction procedure and labor requirements for the extraction procedure. Statistical analyses and gene ontology (GO) classification were employed to further highlight commonalities and differences between the three extraction methods. Protein identities were validated using known protein standards subject to the same shotgun nLC-MS/MS treatment. Breed differences are also discussed.

## Materials and methods

### Milk collection and skim milk recovery

**Figure 1:**
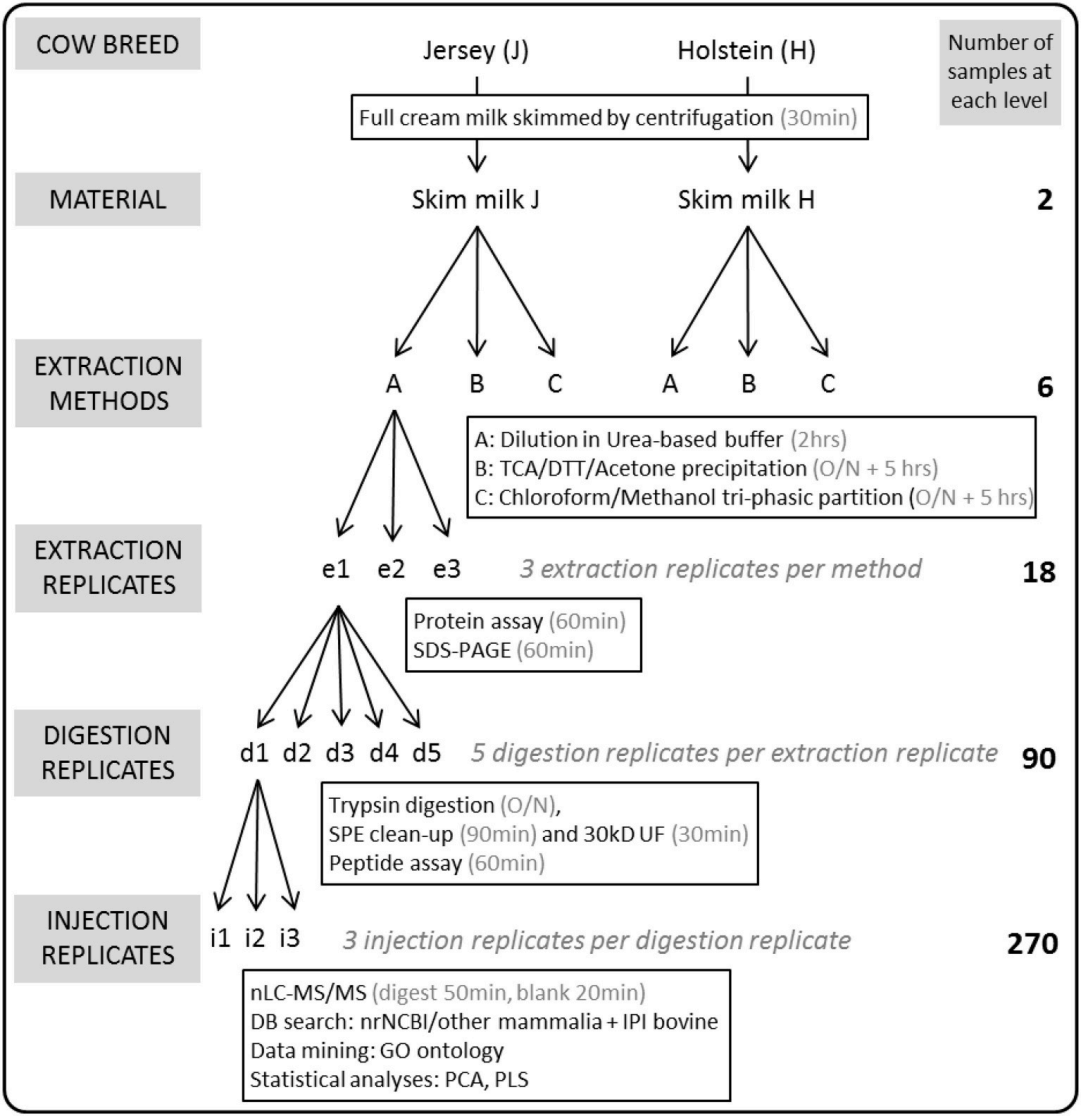
**Overview of the experimental workflow**. Two full cream milk samples were collected from bulk tanks containing the milk of the whole herd of Holstein-Friesian or Jersey cows milked on that particular day. Following centrifugation of the milk to eliminate the cream, proteins were extracted from skim milk in triplicates (e1-e3) using methods A (urea), B (TCA/acetone), or C (methanol/chloroform). All 18 protein extracts were separated using SDS-PAGE, and their protein concentrations obtained in triplicates using the BCA assay. One hundred microgram proteins of each of the 18 extracts were trypsin-digested using five replicates (d1-d5). All 90 tryptic digests underwent Solid Phase Extraction (SPE) clean-up, ultrafiltration (UF) using a 30 kD MWCO; peptide concentrations were obtained using the BCA assay. One hundred nanogram peptides of each of the 90 digests were randomly injected for nLC-MS/MS analysis in triplicates (i1-i3) thus generating 270 MS result files.

Multiparous Holstein-Friesian cows (coded H) were monitored at Ellinbank Research Centre (Victoria, Australia). Jersey cows (coded J) were kept at Wallacevale (Victoria, Australia). The animals were cared for in accordance with the Australian Code of Practice for the Care and Use of Animals for Scientific Purposes (www.nhmrc.gov.au). DeLaval proportional samplers (DeLaval International, Tumba, Sweden) were used to collect a sample of milk from each cow at each milking. Cows were milked twice daily, at 6:00 and 15:00, and milk was bulked into containers. A 50 mL aliquot of bulk milk samples from Jersey cows and from Holstein-Friesian cows were separately collected on 6, November 2014 and stored on ice at the respective dairy farms and during transport. A total of 440 Holstein-Friesian cows contributed to the vat on that date and cows averaged 139 days in milk. A total of 215 Jersey cows contributed to the vat on that date and cows averaged 140 days in milk. Three 2.0 mL milk samples were aliqoted from each bulk sample and stored at −80°C until use. The experimental design is outlined in Figure 1.

Milk samples were skimmed as follows. Frozen full cream milk samples (2.0 mL per tube) were left to thaw at 4°C. Tubes were centrifuged at 4500 rpm for 30 min at 4°C. The skim milk in between the fat layer and the pelleted cells was pipetted (ca. 1.7 mL) and transferred into a fresh 2 mL tube, and this sample immediately underwent extraction.

### Protein extraction methods

Figure 1 outlines the experimental design. Three extraction methods were tested on skim milk samples in triplicates (coded e1 to e3), thus yielding 18 protein extracts.

#### Method A (urea)

The skim milk sample was split into 3 x 0.5 mL aliquots in 2.0 mL tubes. An equal volume (0.5 mL) of Solubilisation Buffer [SB: 6 M urea, 10 mM DTT, 10 mM Tris-HCl pH 8.0, 75 mM NaCl, 0.05% SDS (w:w:v:w:w) in H_2_O] was added and the mixture was vortexed for 1 min. The tubes were incubated at 30°C for 60 min. A 1 M iodoacetamide (IAA) solution was added to reach a final 20 mM concentration and tubes were left to incubate at room temperature in the dark for 60 min. The tubes were centrifuged at 13,000 rpm for 5 min at room temperature. Protein extracts (hereafter named A) were stored at −80°C until use.

#### Method B (TCA/acetone)

The skim milk sample was split into 3 × 0.5 mL aliquots in 2.0 mL tubes. A volume of 1.5 mL 10% TCA, 10 mM DTT in ice-cold acetone (w:w:v) was added which produced a precipitate. The tubes were then vortexed for 1 min and incubated overnight at −20°C for precipitation. The tubes were centrifuged for 10 min at 13,000 rpm and −6°C. The supernatants were discarded. A volume of 1.5 mL 10 mM DTT in ice-cold acetone (w:v) was added. Pellets were first broken down using a spatula and further pulverized by vortexing the tubes for 1 min. The tubes were incubated at −20°C for 60 min, and then centrifuged for 10 min at 13,000 rpm and −6°C. The supernatants were discarded. Pellet washing was repeated once more. The pelleted proteins were dried under vacuum in a Speedvac Concentrator (SPD2010 model, Savant) without heat for 60 min and fully resuspended in 0.5 mL SB by vortexing. A 1 M IAA solution was added to reach a final 20 mM concentration and tubes were left to incubate at room temperature in the dark for 60 min. Protein extracts (hereafter named B) were stored at −80°C until use.

#### Method C (methanol/chloroform)

The skim milk sample was split into 3 × 0.5 mL aliquots in 50 mL tubes. A phase separation extraction procedure adapted from Taylor and Savage (2006) was performed. Briefly, 7.5 mL of chloroform in methanol (1:2) (v:v) was added to the skim milk aliquot and the mixture was vortexed for 1 min. Chloroform (5.0 mL) was added and the mixture vortexed for 1 min. NaCl solution [2.0 mL, (1:10) (w:v)] was added and the mixture vortexed for 1 min. This produced a triphasic solution with a protein interphase. To maximize phase separation, the tube was centrifuged at 5100 rpm for 30 min at room temperature using a swing bucket rotor. Both upper and lower phases were carefully discarded and the remaining wet interphase was transferred into a fresh 1.5 mL tube. The interphase was dried under vacuum using a SpeedVac Concentrator for 60 min. The dry interphase was resuspended by adding 0.5 mL of a SB and letting the interphase slowly reabsorb SB during an overnight incubation at 4°C. Resupension of the interphase was finalized by vortexing for 30 min using a Multi Tube Vortex Mixer (MTV1 model, Ratek) at full speed at room temperature. A 1 M IAA solution was added to reach a final 20 mM concentration and tubes were left to incubate at room temperature in the dark for 60 min. Protein extracts (hereafter named C) were stored at −80°C until use.

### Protein assay

The protein concentrations of the skim milk aliquots and milk extracts (1:10 dilution) were assessed in duplicate using the Microplate BCA protein assay kit (Pierce) following the manufacturer's instructions which are based on the method developed by Smith et al. (1985). Bovine Serum Albumin (BSA) was used a standard. For each extract, the recovery rate of protein extraction was computed as a percentage of skim milk protein concentration.

### SDS-PAGE

The complexity of milk protein patterns were initially analyzed by SDS-PAGE using pre-cast NuPAGE® Novex gels (4–12% bis-tris acrylamide, 1 mm, 8 × 8 cm, 10 lanes, Life Technologies). A volume of skim milk or protein extract corresponding to 50 μg of proteins was loaded per lane. Samples were diluted with the loading buffer (0.5 M DTT added to 4X NuPAGE LDS Sample Buffer, Life Technologies) to reach a final 20 μL volume and heated at 70°C for 10 min. Samples were loaded on the gels and run using MOPS-SDS running buffer (50 mM MOPS, 50 mM Tris Base, 0.1% SDS 1 mM EDTA, pH 7.7 in H_2_O) for 35 min at 300V at 4°C until the blue front reached the bottom of the gel. Novex SeeBlue^R^ Pre-Stained Standard (Life Technologies) was loaded in the first lane of each gel to estimate the molecular weight (MW) of the milk proteins and account for gel to gel variation.

Gels were stained using a Colloidal Coomassie Blue (CCB) method as follows. Gels were incubated at room temperature for 48 h on an orbital shaker in 200 mL of CCB solution (2% phosphoric acid, 18% ethanol, 15% ammonium sulfate, 1% Brilliant Blue G250 (v:v:w:w) in H_2_O). Gels were rinsed twice for 30 min in H_2_O and scanned using a CanoScan 8800F scanner (Canon).

### In-solution protein digestion using trypsin protease

Digestions were performed five times (coded d1 to d5) on each protein extract, thus yielding 90 peptide digests. An aliquot corresponding to 100 μg of milk proteins was used for protein digestion as follows. The DTT-reduced and IAA-alkylated proteins were diluted six times using 50 mM ammonium bicarbonate (ABC) to decrease the urea molarity below 1 M. Trypsin protease (Sequencing Grade Modified Trypsin, 20 μg aliquots, Promega) was carefully solubilised in 1 mL of the resuspension buffer supplied by the manufacturer (50 mM acetic acid) and incubated for 15 min at 30°C to maximize its activity. An aliquot of trypsin was added and gently mixed with the milk proteins so as to reach a 1:50 ratio of trypsin:milk proteins. The mixture was left to incubate overnight (19 h) at 37°C in the dark. The digestion reaction was stopped by lowering the pH of the mixture using a 10% formic acid (FA) in H_2_O (v:v) to a final concentration of 1% FA.

### Tryptic digest cleaning, assay, and dilution

The 90 tryptic digests were desalted using solid phase extraction (SPE) cartridges (Sep-Pak C18 1cc Vac Cartridge, 50 mg sorbent, 55–105 μm particle size, 1 mL, Waters) by gravity as follows. The SPE cartridges were conditioned by running 1 mL of 80% acetonitrile (ACN):0.1% FA in H_2_O (v:v:v) and then washed using 1 mL of 0.1% FA in H_2_O (v:v). The tryptic digests were loaded onto the cartridges and washed using 1 mL of 0.1% FA in H_2_O (v:v). Peptides were eluted using 1 mL of 80% ACN:0.1% FA in H_2_O (v:v:v) into a fresh 1.5 mL tube. The eluent's volume (1.00 mL) was reduced to 0.18 mL using a Speedvac Concentrator without heat, thereby ensuring the complete evaporation of the ACN.

Undigested milk proteins were filtered out using ultrafiltration (UF) devices (MWCO 30 kD, 0.5 mL, Amicon Ultra-0.5 centrifugal filter device, Millipore). The filtrates were collected and the peptide concentration was assessed using the Microplate BCA protein assay kit (Pierce), as per the manufacturer's instructions albeit excluding the compatibility reagent step. Bovine Serum Albumin (BSA) was used a standard.

An aliquot corresponding to 10 μg of peptide digest was diluted with 0.1% FA in H_2_O (v:v) to reach a final volume of 100 μL (0.1 μg/μL). The diluted peptide mixture was transferred into a 100 μL glass insert placed into a glass vial. The vials were positioned into the autosampler at 4°C until MS analyses.

### Nano-liquid chromatography (nLC)-electrospray ionization (ESI) tandem MS (MS/MS) analyses

The nLC-ESI-MS/MS analyses were performed in triplicates (coded i1 to i3) thus yielding 270 MS files. The coding of the samples at the last stage follows the pattern breed_method_extraction-replicate_digestion-replicate_injection-replicate (e.g., JAe1d1i1 stands for Jersey breed_method A/extraction-replicate 1_digestion-replicate 1_injection-replicate 1). The injection order was randomized to minimize systematic error including chromatographic drift or suppression effects. Chromatographic separation of the tryptic peptides was performed by reverse phase (RP) using an Ultimate 3000 RSLCnano System (Dionex). A 1 μL aliquot (0.1 μg peptide) was loaded using a full loop injection mode onto a trap column (Acclaim PepMap100, 75 μm × 2 cm, C_18_ 3 μm 100 Å, Dionex) at a 3 μL/min flow rate and switched onto a separation column (Acclaim PepMap100, 75 μm × 15 cm, C_18_ 2 μm 100 Å, Dionex) at a 0.4 μL/min flow rate after 3 min. The column oven was set at 30°C. Mobile phases for chromatographic elution were 0.1% FA in H_2_O (v:v) (phase A) and 0.1% FA in ACN (v:v) (phase B). Ultraviolet (UV) trace was recorded at 215 nm for the whole duration of the nLC run. A linear gradient from 3 to 40% of ACN in 35 min was applied. Then ACN content was brought to 90% in 2 min and held constant for 5 min to wash the separation column. Finally, the ACN concentration was lowered to 3% over 0.1 min and the column re-equilibrated for 5 min. On-line with the nLC system, peptides were analyzed using an Orbitrap Velos hybrid ion trap-Orbitrap mass spectrometer (Thermo Scientific). Ionization was carried out in the positive ion mode using a nanospray source. The electrospray voltage was set at 2.2 kV, and the heated capillary was set at 280°C. Full MS scans were acquired in the Orbitrap Fourier Transform (FT) mass analyser over a normal range of 300–2000 *m/z* with 60,000 resolution in profile mode. MS/MS spectra were acquired in data-dependent mode. The 20 most intense peaks with charge state ≥ 2 and a minimum signal threshold of 10,000 were fragmented in the linear ion trap using collision-induced dissociation (CID) with a normalized collision energy of 35%, 0.25 activation Q, and activation time of 10 ms. The precursor isolation width was 2 *m/z*. Dynamic exclusion was enabled, and peaks selected for fragmentation more than once within 10 s were excluded from selection for 30 s. Blanks (1 μL of mobile phase A) were injected in between each peptide digest and analyzed over a 20 min nLC run to further clean the C_18_ separation column, and minimize carry-over.

### Database search for protein identification

Database searching of the 270 MS.RAW files was performed in Proteome Discoverer 1.4 with MASCOT 2.4.1 against both the non-redundant (nr) National Center for Biotechnology Information (NCBI) database with taxonomy as mammalia (2,578,153 entries, released on 7 November 2014, 68th release) and the International Protein Index (IPI) bovine database (23,841 entries, last modified on 4 April 2014, http://www.uniprot.org/proteomes).

The database searching parameters specified trypsin as the digestion enzyme and allowed up to two missed cleavages. The precursor mass tolerance was set at 10 ppm, and fragment mass tolerance set at 0.5 Da. Carbamidomethylation (C) was set as a static modification. Oxidation (M), phosphorylation (STY), conversion from Gln to pyro-Glu (N-term Q) and Glu to pyro-Glu (N-term E), and deamination (NQ) were set as dynamic modifications. The target decoy peptide-spectrum match (PSM) validator was used to estimate false discovery rates (FDR). At the peptide level, peptide confidence value set at high was used to filter the peptide identification, and the corresponding FDR on peptide level was less than 1%. At the protein level, protein grouping was enabled.

The mass spectrometry proteomics data have been deposited to the ProteomeXchange Consortium (Vizcaíno et al., 2014) via the PRIDE partner repository with the dataset identifier PXD002529.

The amino acid sequences of the proteins annotated as “Uncharacterized” were searched using the Basic Local Alignment Search Tool (BLAST) tool of UniProt database (http://www.uniprot.org/blast/) with the default parameters except for the target database which was set at “Mammals.” The best hit is indicated in brackets in Table 1.

**Table 1:** **List of protein accessions identified in the different milk extracts, along with their description, their coverage (percentage of the database protein sequence covered by matching peptides) across samples, the number of unique peptides (distinct peptide that match to a single protein entry within the search database), the occurrence (%) in the three methods and the two breeds**.

**No**	**Description**	**gi Accession**	**IPI accession**	**Σcoverage (%)**	**Σ# Unique Peptides**	**Occurrence^a^ in method A (%)**	**Occurrence^a^ in method B (%)**	**Occurrence^a^ in method C (%)**	**Occurrence^a^ in breed H (%)**	**Occurrence^a^ in breed J (%)**
1	11 kDa protein		IPI00843089.3	52.3	22	100	100	100	100	100
2	**Actin 1**	4501885	IPI00698900.1	16.5	520	72	67	40	47	**73**
3	Alpha-1-acid glycoprotein	94966811	IPI00691212.1	33.7	10	97	100	27	77	75
4	**Alpha-1-antiproteinase**	27806941	IPI00695489.1	24.5	10	97	94	85	**98**	87
5	**Alpha-1B-glycoprotein**	114053019	IPI00692686.1	13.3	6	9	39	35	**41**	13
6	**Alpha-2-HS-glycoprotein**	27806751	IPI00707101.1	19.2	8	86	74	65	**90**	62
7	Alpha-lactabumin	119393699		30.8	3	95	0	55	50	51
8	Alpha-lactalbumin	505782142		9.9	1	7	0	0	5	0
9	Alpha-lactalbumin	11036986		18.1	2	100	100	100	100	100
10	Alpha-lactalbumin	27805979	IPI00717424.1	31.0	53	55	0	0	18	21
11	Alpha-lactalbumin-like isoform X3	585153938		12.3	3	100	0	0	36	34
12	Alpha-S1-casein	159793227		15.8	1	100	100	100	100	100
13	Alpha-S1-casein	299676		64.7	1	0	1	0	0	1
14	**Alpha-S1-casein**	162650		14.0	1	22	12	17	**22**	11
15	Alpha-S1-casein isoform X2	528953236		41.3	47	100	100	100	100	100
16	Alpha-S1-casein isoform X3	528953238		42.2	47	100	100	100	100	100
17	Alpha-S1-casein isoform X6	528953244		30.2	5	100	100	100	100	100
18	Alpha-S1-casein isoform X7	528953246		49.2	47	100	100	100	100	100
19	Alpha-S2-casein	27806963	IPI00698843.1	59.5	29	0	4	10	5	4
20	Alpha-S2-casein	147744646		24.1	1	100	100	100	100	100
21	**Angiogenin-1**	118151356	IPI00726982.1	31.8	5	76	39	74	**69**	57
22	**Antibody Blv5b8**	513137425		9.5	5	51	6	35	**41**	21
23	Anti-hiv llama vhh antibody a12	380258837		6.3	1	0	0	1	0	1
24	Apolipoprotein A-I preproprotein	75832056	IPI00715548.1	38.1	56	99	0	99	67	63
25	**Apolipoprotein A-II**	114052298	IPI00688815.2	16.0	5	25	0	0	**14**	3
26	Apolipoprotein A-IV	82697389	IPI00695965.1	18.7	19	36	0	4	12	16
27	Apolipoprotein E	27806739	IPI00712693.1	7.3	12	75	0	15	30	32
28	ATP-binding cassette sub-family G member 2	112817615	IPI00690408.5	7.9	80	100	0	81	62	59
29	**Beta-1,4-galactosyltransferase 1**		IPI00760476.26	5.1	2	90	29	0	**54**	29
30	Beta-1,4-galactosyltransferase 1	116241263	IPI00685910.2	10.0	29	0	0	5	3	0
31	Beta-2-microglobulin	41386683	IPI00686769.1	23.7	22	100	82	97	96	90
32	Beta-casein	555980347	IPI00697085.1	23.7	8	0	0	81	27	23
33	Beta-casein	83406093		23.7	45	0	100	0	32	36
34	Beta-casein variant I	38231527		35.9	2	35	0	0	12	13
35	Beta-lactoglobulin	54037712		37.0	22	1	0	0	0	1
36	Beta-lactoglobulin	562890035		30.0	1	100	100	100	100	100
37	Beta-lactoglobulin	87196497	IPI00699698.1	41.0	18	5	0	19	6	10
38	Beta-lactoglobulin	223165		47.5	19	100	0	100	68	64
39	Beta-lactoglobulin	49259423		47.5	15	100	0	0	36	34
40	Beta-lactoglobulin	378947940		25.8	1	100	100	0	68	70
41	Beta-lactoglobulin A	2194088		47.5	22	100	0	0	36	34
42	**Beta-lactoglobulin-like**	593730995		9.4	1	22	0	0	2	**13**
43	Butyrophilin subfamily 1 member A1	3183510	IPI00708535.1	28.1	335	100	100	100	100	100
44	Cathelicidin-1	27807341	IPI00718108.1	10.3	10	6	0	1	5	0
45	**Cathelicidin-4**	27807337	IPI00686754.1	11.1	1	5	13	1	**12**	1
46	**CD5L protein**		IPI00867131.2	14.1	23	25	0	81	22	**45**
47	CD81 antigen	78042548	IPI00685617.1	6.8	9	89	8	0	37	31
48	Cell division control protein 42 homolog isoform 1	4757952	IPI00704257.2	5.2	76	19	0	33	19	15
49	Chemokine (C-X-C motif) ligand 3	114050915	IPI00721750.2	14.4	9	95	0	0	34	33
50	Clusterin preproprotein	27806907	IPI00694304.1	18.0	53	97	95	99	95	98
51	Complement C3	602697202		7.9	86	100	100	100	100	100
52	Complement C3	124056491	IPI00713505.2	34.0	152	100	0	0	36	34
53	Cysteine-rich secretory protein 3	118601862	IPI00715999.1	23.4	7	99	78	90	91	87
54	dnaJ homolog subfamily B member 9	300795871	IPI01000889.1	17.9	89	0	20	13	8	13
55	dnaJ homolog subfamily C member 3	27807457	IPI00693007.1	3.4	14	0	0	1	1	0
56	Dystroglycan	27806449	IPI00707359.1	1.8	21	0	91	1	32	30
57	Endopin 2B	38683423	IPI01017613.1	16.6	7	92	0	0	35	29
58	**Epididymal secretory protein E1**	27806881	IPI00711862.1	32.9	8	59	80	8	**62**	38
59	**Fab Pgt123 Hiv-1 Neutralizing Antibody**	491668737		5.2	1	56	13	0	**34**	13
60	Factor XIIa inhibitor	27807349	IPI00710025.1	15.4	7	100	68	71	82	78
61	Fatty acid synthase	426239165		1.2	11	8	0	0	1	5
62	**Fatty acid-binding protein**	507563106		14.3	40	99	40	100	**94**	64
63	Fatty acid-binding protein	27805809	IPI00691946.2	47.4	109	80	0	9	34	28
64	**Fibrinogen alpha chain**	75812954	IPI00691819.1	8.9	7	95	28	63	**77**	48
65	**Fibrinogen beta chain**	218931172	IPI00709763.5	11.7	24	27	53	49	**49**	37
66	**Fibroblast growth factor-binding protein 1**	27805911	IPI00704023.1	11.5	5	94	40	92	**87**	63
67	Fibronectin isoform X10	528940100	IPI01028178.1	0.6	484	0	0	3	0	2
68	**Folate receptor alpha**	330688394	IPI01017673.1	9.1	4	76	74	0	**59**	44
69	Gelsolin isoform b	296484315	IPI01017675.1	1.8	190	0	11	0	2	5
70	Glutamyltranspeptidase 1 gamma	329664306	IPI00705565.2	6.7	4	10	2	0	2	7
71	Glycosylation-dependent cell adhesion molecule 1	27807339	IPI00716366.1	50.3	39	100	100	100	100	100
72	GTP binding protein Rab1a	15281851	IPI00883580.1	17.0	118	0	1	37	16	8
73	hCG1791766 isoform CRA_a	119626014		13.7	2	0	18	47	22	20
74	Heat shock cognate 71 kDa protein	548494982	IPI00708526.2	5.9	656	14	0	0	4	6
75	Heat shock-related 70 kDa protein 2	148887197	IPI00710052.1	1.7	1	0	1	19	8	5
76	Hedgehog interacting protein-like 2-like	76669880	IPI00700055.2	11.5	10	0	80	96	60	54
77	Hemopexin	77736171	IPI00690198.4	3.5	6	2	0	0	2	0
78	Heparin cofactor 2	157280001	IPI00688367.1	2.6	6	0	5	0	1	2
79	**Histatherin**	242347807	IPI00944405.1	39.7	2	13	12	88	**44**	28
80	Hornerin	28557150		4.1	6	0	0	1	0	1
81	**Ig anti-HIV-1**	612405504		12.6	1	1	0	59	13	**25**
82	Ig J chain	32401410	IPI00701295.1	29.9	20	99	100	99	98	100
83	Ig lambda light chain	15088675		21.3	10	100	0	100	68	64
84	Ig lambda light chain constant region 2 allotypic variant IGLC2b	343197004		30.2	1	98	100	0	67	69
85	Ig lambda light chain constant region 3 allotypic variant IGLC3c	343197026		52.8	3	100	100	100	100	100
86	**Ig lambda-1 variable region**	42759882		13.9	2	0	0	19	1	**11**
87	Ig lambda-like polypeptide 1-like		IPI01002118.1	23.3	8	100	0	100	68	64
88	Ig light chain	310893433		16.7	3	91	62	77	80	74
89	Ig light chain	310893435		61.4	16	100	0	100	68	64
90	Ig light chain variable region	5802436		11.5	1	100	100	100	100	100
91	Ig light chain variable region	2323400		41.8	12	0	4	3	4	0
92	Ig light chain variable region	482673169		9.4	15	16	6	0	10	6
93	Ig light chain, lambda gene cluster	92096965	IPI00867205.1	20.1	1	0	100	0	32	36
94	IgG1 heavy chain constant region	7547266		16.4	9	98	95	99	98	97
95	**IgG2a heavy chain constant region**	1699167		12.9	2	48	5	45	**42**	23
96	IGK protein	115545495	IPI00724838.2	10.0	10	0	2	99	34	29
97	IgM heavy chain constant region	2232299		22.7	53	100	100	100	100	100
98	Inhibitor of carbonic anhydrase-like	470655576		5.2	7	0	6	0	3	1
99	Isocitrate dehydrogenase cytoplasmic	75056526	IPI00702781.2	12.1	5	0	0	14	6	2
100	Isocitrate dehydrogenase cytoplasmic	75832090	IPI01028293.1	25.9	148	77	0	0	28	26
101	Kappa-casein	8099324		40.6	33	1	6	13	5	8
102	Kappa-casein	162807		42.4	42	100	100	100	100	100
103	Kappa-casein	1705608		24.0	135	100	100	100	100	100
104	Kappa-casein	284027124		17.3	133	100	54	100	88	81
105	Kappa-casein	315143016		36.8	67	58	26	24	32	41
106	Kappa-casein	428755215		13.6	2	100	100	100	100	100
107	Kappa-casein	428755219		33.3	66	99	100	100	99	100
108	Kappa-casein	284626		58.5	1	100	100	100	100	100
109	Kappa-casein	2493514		20.5	10	100	100	100	100	100
110	Kappa-casein	251198		100.0	1	100	100	100	100	100
111	Kappa-casein	1594249		9.0	2	100	100	0	68	70
112	Kappa-casein A	229416		57.1	33	100	100	100	100	100
113	**Kininogen-1**	125505	IPI00701166.1	11.6	13	99	0	26	**50**	36
114	**Kininogen-2**	490	IPI00718535.1	10.4	4	0	53	0	**29**	7
115	Kininogen-2 isoform X1	528936694		14.7	17	99	0	0	35	34
116	**Lactadherin**	2494285	IPI00689035.1	33.3	55	100	62	100	**100**	75
117	Lactoperoxidase	27806851	IPI00716157.1	26.1	144	36	0	36	23	25
118	Lactoperoxidase	16740670		5.1	12	100	100	100	100	100
119	Lactotransferrin	589925430		4.0	24	100	100	100	100	100
120	Lactotransferrin	30794292	IPI00710664.1	53.1	134	98	0	0	35	33
121	Lethal(3)malignant brain tumor-like protein 4	505792423		2.5	1	1	0	1	2	0
122	Leucine proline-enriched proteoglycan (leprecan) 1	507937306		1.9	1	1	0	1	1	1
123	Lipopolysaccharide-binding protein	84579853	IPI00730056.1	2.3	5	2	0	0	1	1
124	Lipoprotein lipase	115497164	IPI00692291.3	22.0	152	100	99	100	100	99
125	Lymphocyte antigen 96	114051704	IPI00689371.2	6.3	9	3	0	0	1	2
126	Lymphocyte-specific protein 1 isoform X6	587003867		5.8	4	3	0	0	1	2
127	Mammaglobin-A	155372309	IPI00711254.1	14.1	1	10	0	0	7	0
128	**Mammary serum amyloid A3.2**	347300329	IPI01017597.1	9.2	11	17	1	0	0	**13**
129	Monocyte differentiation antigen CD14 isoform X1	528956860	IPI00686931.2	22.7	25	82	0	68	47	52
130	Mucin-1	41386778	IPI00706283.1	3.1	13	100	7	8	43	37
131	Mucin-15	41386723	IPI00716220.1	7.3	10	99	100	94	97	98
132	Myristoylated alanine-rich C-kinase substrate	148872484	IPI00760436.1	6.0	6	0	9	0	6	0
133	Neutrophil gelatinase-associated lipocalin isoform X3	528912092	IPI00685784.3	30.5	11	28	95	74	65	66
134	Nucleobindin-1	115497814	IPI00722271.2	28.1	117	100	0	38	48	46
135	Nucleobindin-2	115496067	IPI00696729.2	15.2	73	0	100	0	32	36
136	Olfactory receptor 2S2-like	724801196		2.2	1	0	4	1	2	1
137	Osteopontin isoform X1	528952550	IPI00691887.2	31.7	71	99	100	100	99	100
138	Pancreatic elastase inhibitor	861514		66.7	1	10	0	0	1	6
139	Pancreatic secretory granule membrane major glycoprotein GP2	341572597		23.0	84	100	96	100	100	98
140	**Peptidoglycan recognition protein 1**	27808640	IPI00701640.1	13.7	5	0	15	63	**37**	13
141	Peptidyl-prolyl cis-trans isomerase A	47523764	IPI00697285.3	16.5	101	6	2	8	4	6
142	Peptidyl-prolyl cis-trans isomerase B	555965067	IPI00702098.4	6.0	85	31	0	0	14	8
143	Peptidyl-prolyl cis-trans isomerase FKBP1A	78365305	IPI00698916.2	25.0	98	7	0	0	2	2
144	Perilipin-2 isoform X2	528958213	IPI01017444.1	38.0	96	100	100	100	100	100
145	Platelet glycoprotein 4	521258696	IPI00710204.3	15.5	106	100	100	100	100	100
146	Polymeric immunoglobulin receptor	3914346	IPI00696714.1	30.0	28	100	0	100	68	64
147	Polymeric immunoglobulin receptor	296479365		23.9	21	100	100	100	100	100
148	Polyubiquitin	2627129		21.0	380	0	79	0	24	29
149	Prosaposin	27806447	IPI00718311.3	5.0	54	25	0	0	11	6
150	Prostaglandin-H2 D-isomerase	27807521	IPI00709683.1	7.3	6	65	94	100	85	87
151	Protein CREG1	115495283	IPI00702458.2	20.4	11	78	0	13	30	33
152	Protein HP-25 homolog 1	114050753	IPI00724799.2	7.1	3	1	0	0	1	0
153	**Protein HP-25 homolog 2**	114052108	IPI00700655.3	14.9	3	7	2	40	**27**	4
154	Protein inturned	617602241		1.3	1	1	0	0	1	0
155	Protein OS-9	77735409	IPI00706896.1	4.5	2	5	0	1	2	2
156	Prothrombin	135806	IPI00710799.1	2.2	7	6	1	0	2	3
157	Ras-related protein Rab-11A	84000297	IPI00695221.3	11.1	82	1	0	0	1	0
158	Ras-related protein Rab-18	115495023	IPI00691826.3	7.3	123	3	0	0	1	2
159	Rhophilin-2-like protein RHPN2P1	190360225		2.4	2	0	1	0	1	0
160	Ribonuclease 4	32363484	IPI00697112.1	9.2	1	0	81	0	30	25
161	Ribonuclease 4	95006989	IPI00760446.1	28.6	4	31	0	3	12	11
162	Ribonuclease pancreatic	133210		10.5	100	0	19	0	3	10
163	Secretoglobin family 1D member	118150406	IPI00824879.1	21.6	4	100	100	100	100	100
164	Selenium-binding protein 1		IPI00718529.1	1.7	1	8	0	3	3	4
165	Selenoprotein	148680082		13.0	69	45	0	9	23	14
166	Serotransferrin	2501351	IPI00690534.1	45.5	61	100	95	100	100	97
167	**Serpin A3-1**	160332365	IPI00700622.4	12.9	32	100	0	77	**67**	51
168	Serum albumin	1351907	IPI01028455.1	65.1	40	100	61	100	83	90
169	Sodium-dependent phosphate transport protein 2B	27807195	IPI00703813.1	5.9	7	92	0	0	35	29
170	Sulfhydryl oxidase 1	156120795	IPI00867237.1	19.1	11	98	27	97	75	72
171	Superoxide dismutase	134289051	IPI00847121.1	11.8	73	0	1	0	0	1
172	Sushi repeat-containing protein SRPX	507969850		3.9	3	0	2	6	2	4
173	Tetranectin	114051137	IPI00717369.1	6.9	5	89	0	1	34	29
174	Transforming protein RhoA	10835049	IPI00688998.3	6.2	49	0	0	1	1	0
175	Transthyretin	27806789	IPI00689362.1	34.7	24	0	91	77	59	50
176	Uncharacterized protein^b^ (P02666 Beta-casein 71% ident.)		IPI00712994.3	33.5	5	18	0	0	7	6
177	Uncharacterized protein^b^ (F1MGU7 Fibrinogen gamma-B chain 100% ident.)		IPI00843209.1	17.2	16	99	0	0	35	34
178	**Uncharacterized protein^b^ (A5PK72 Uncharacterized protein 92% ident.)**		IPI01017618.1	20.6	10	42	0	0	10	**20**
179	Uncharacterized protein^b^ (A0A0A0MP92 Serpin A3-7 98% ident.)		IPI00971595.1	21.1	4	1	0	6	5	0
180	**Uncharacterized protein^b^ (Q2HJI6 Granulin 99% ident.)**		IPI00904166.1	6.3	99	98	0	41	**57**	37
181	Uncharacterized protein^b^ (F1MLW7 Uncharacterized protein 97% ident.)		IPI00838162.2	20.9	1	0	100	0	32	36
182	Uncharacterized protein LOC524810^b^ (A5D7Q2 Uncharacterized protein 100% ident.)	326937675	IPI00852509.1	10.9	6	100	100	100	100	100
183	**Vitamin D-binding protein**	78369364	IPI00823795.1	8.0	20	38	0	0	**21**	6
184	Xanthine dehydrogenase/oxidase	296482686		15.6	16	0	100	0	32	36
185	Xanthine dehydrogenase/oxidase	109940048	IPI00695367.3	14.1	16	100	0	100	68	64
186	Zinc-alpha-2-glycoprotein	77735615	IPI00698993.1	11.4	2	1	100	100	64	67

*Proteins whose occurrence varies more than 10% across breeds are highlighted in bold*.

a *Occurrence is defined as the ratio of the number of samples in which the protein was identified over the total number of samples in the method or breed expressed in percent*.

b *Amino acid sequence of uncharacterized proteins were blasted against uniprot database. Best hit is indicated in brackets with the percentage of identity*.

### Statistical analyses

The 270 MS.RAW files were post-processed using Genedata Expressionist Refiner 8.1 as follows. The chemical noise was substracted by smoothing chromatograms over 50 scans retention time (RT) window. The intensities were put onto a common *m/z*-RT adaptive grid over 10 scans. Chromatograms were then aligned using a Pairwise Alignment Based Tree scheme with a 50 scan interval. Chromatograms were averaged using a mean method. Chromatographic peaks were detected using a 1 min summation window and a curvature-based peak detection method. Chromatogram istopes were clustered using a Peptide Isotope Shaping method with 0.05 min RT tolerance and 0.01 Da *m/z* tolerance. A reference grid was then applied and the reference peaks extracted. A MS/MS consolidation node was performed by filtering MS/MS not in cluster on the highest Total Ion Chromatogram (TIC). Identification results from Proteome Discoverer were then imported and peaks annotated. The resulting peaks were exported to Genedata Expressionist Analyst 8.1 for further statistical analyses.

In Analyst, peaks were normalized using an Intensity Drift Normalization method using the randomized injection order. Principal component analyses (PCA) were applied to the normalized peaks using a covariance matrix of row means with 50% valid values. Partial Least Squares analyses (PLS) were performed on row means using the cow breed as a response, three latent factors, and 50% valid values.

### Gene ontology (GO) classification

The database search produced two types of accessions: Gene Index (gi) and IPI. International Protein Index accessions were converted to gi accession numbers using the gi2ipi.xrefs file available at the European Bioinformatics Institute website (ftp.ebi.ac.uk/pub/databases/IPI/last_release/current). Gene Ontology terms were retrieved on-line from all gi accessions using UniProtKB Retrieve ID/Mapping tool (http://www.uniprot.org/uploadlists/). Results were exported into Microsoft Excel 2010 and charts generated.

### Validation of protein identifications using known standards

In order to confirm the identities of some of the proteins identified in this study either commonly across breeds and extraction methods, or displaying qualitative variation across breeds and/or methods, bovine protein standards were purchased from Sigma from bovine wherever possible otherwise from human. If the bovine derived protein was not available the human protein was obtained. The protein standards include: actin from bovine muscle (A3653-1MG, 80% pure), fibrinogen from bovine plasma (F8630-1G, type I-S, 65–85% pure), lactoferrin from bovine milk (L9507-10MG, 85% pure), kininogen low molecular weight from human plasma (K3628-1MG, 95% pure), α-casein from bovine milk (C6780-250MG, 70% pure), β-casein from bovine milk (C6905-250MG, 98% pure), κ-casein from bovine milk (C0406-250MG, 70% pure), α-lactalbumin from bovine milk (L5385-25MG, 85% pure), β-lactoglobulin from bovine milk (L3908-250MG, contains lactoglobulins A and B, 90% pure), albumin from bovine serum (BSA, A7906-10G, 98% pure). These lyophilised protein standards were fully solubilised at a 10 mg/mL concentration in SB which contained 10 mM DTT. After a 60 min incubation at room temperature, a 1 M IAA solution was added to reach a final 20 mM concentration and tubes were left to incubate at room temperature in the dark for 60 min. These individual standards were combined together in equamolarity to make a mix which was duplicated. This mix was used to spike two milk extracts obtained using method A (JAe1 and HAe3) and chosen because their protein concentrations were the closest to those of the standards. Standard mixtures and extracts JAe1 and HAe3, spiked or unspiked, underwent trypsin digestion as described in Section In-Solution Protein Digestion Using Trypsin Protease by pipetting a volume corresponding to 100 μg of proteins. For the milk extracts spiked with the mix, 100 μg of proteins from milk extracts were spiked with 100 μg of proteins from the mix. A 1:50 ratio of trypsin:standards was used. The subsequent clean-up, nLC-MS/MS, database search steps were rigorously performed as described above in Sections Tryptic Digest Cleaning, Assay and Dilution, Nano-Liquid Chromatography (nLC)-electrospray ionization (ESI) tandem MS (MS/MS) analyses, and Database Search for Protein Identification.

## Results

### SDS-PAGE patterns, protein concentrations, number of accessions, and nLC-MS runs

**Figure 2:**
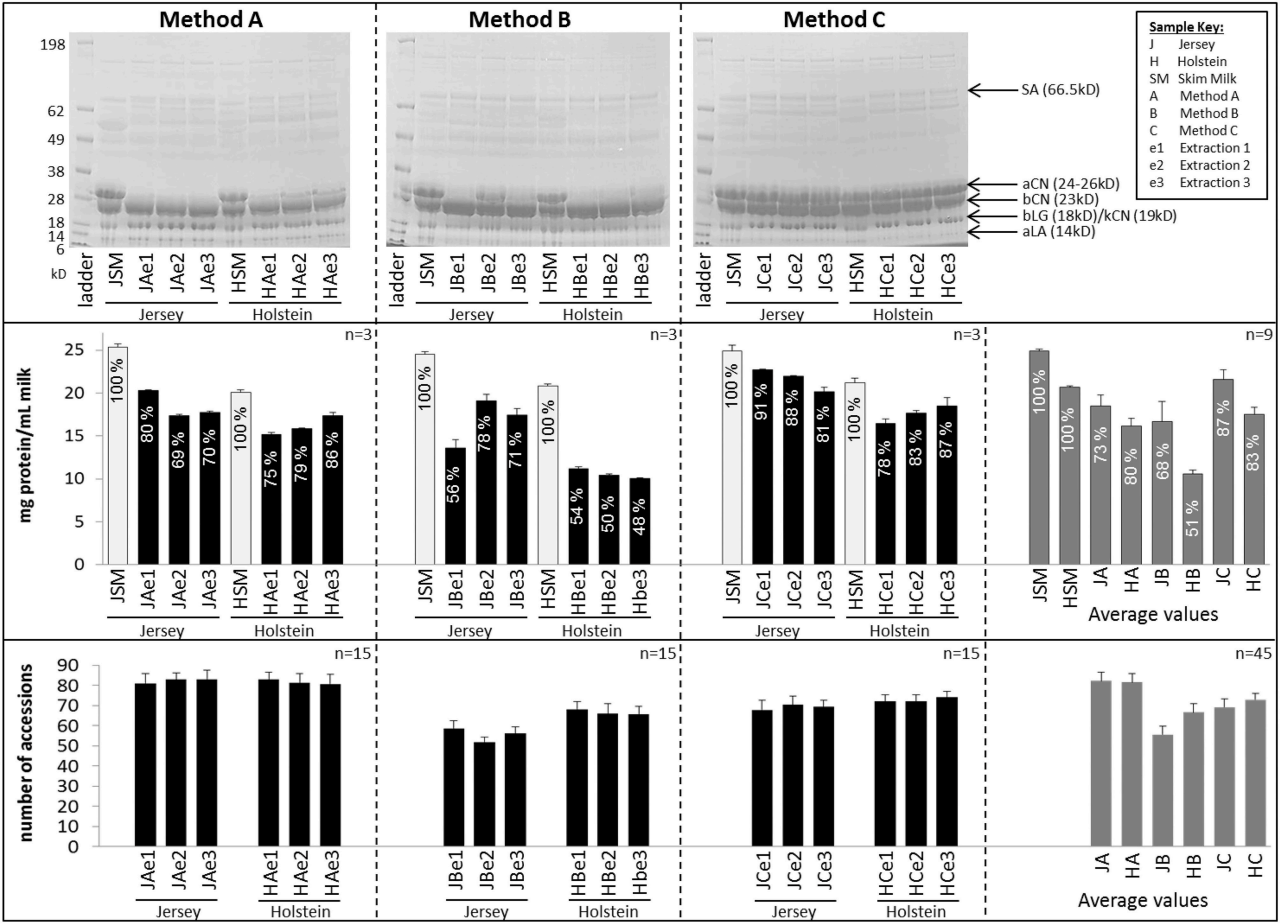
**Comparison of SDS-PAGE patterns (top panel), protein concentration (middle panel), and number of protein accessions identified per sample (bottom panel)**. Error bars are Standard Deviation (SD); the n number displayed at the top right corner of each box represents the number of replicates used for average and SD. Error bars for the protein assay are from the BCA technical triplicates. Error bars for the accession numbers are from 15 replicates (5 digestion replicates × 3 injection replicates). Recovery rates are indicated in percent in the protein assay and are computed relative to protein concentrations in skim milk (SM). SA, Serum Albumin; aCN, alpha-casein; bCN, beta-casein, bLG, beta-lactoglobulin; aLA, alpha-lactalbumin.

Figure 2 displays SDS-PAGE profiles, protein concentrations and number of protein accessions identified for each milk sample across all three sets of protein extracts.

The same amount of proteins was loaded per extract to produce SDS-PAGE profiles, with skim milk as a reference. The electrophoretic patterns were similar from one extraction method to another, albeit extracts C (methanol/chloroform) displayed the best resolution with the sharpest bands. In particular extracts C (methanol/chloroform) were the only ones consistently resolving the very intense 24–26 kD band corresponding to α-caseins, and were therefore more comparable to skim milk profiles than extracts A (urea) and B (TCA/acetone). This band was either missing or very faint on SDS-PAGE profiles of extracts A (urea) and B (TCA/acetone), except for extract JBe2.

Protein assays were performed in triplicate, using skim milk as a reference to compute recovery percentage (%) following protein extraction procedure. Protein concentrations were converted to mg *per* mL of milk. Jersey cow milk had a greater protein concentration (24.9 mg/mL) than Holstein-Friesian cow milk (20.7 mg/mL). This is consistent with the literature which also reports higher concentrations of milk fats in Jersey breed than Holstein-Friesian's (Arnould and Soyeurt, 2009; Capper and Cady, 2012; Jensen et al., 2012b). All methods considered, protein concentrations ranged from 10.1 (HBe3) to 22.7 (JC1) mg/mL. Standard deviation (SD) was less than 10% of the mean. On average, protein concentrations for Jersey breed were 18.5 (±1.3) mg/mL (73%), 16.7 (± 2.3) mg/mL (68%), and 21.6 (±1.1) mg/mL (87%), respectively for extracts A (urea), B (TCA/acetone) and, C (methanol/chloroform). On average, concentrations for Holstein-Friesian breed were 16.1 (±0.9) mg/mL (80%), 10.5 (± 0.5) mg/mL (51%), and 17.5 (±0.8) mg/mL (83%), respectively for extracts A (urea), B (TCA/acetone), and C (methanol/chloroform). Figure 2 shows that method C (methanol/chloroform) yielded the highest protein concentrations substantiated by the highest recovery rate, followed by method A (urea), while method B (TCA/acetone) resulted in the lowest concentrations particularly for Holstein-Friesian breed.

The number of unique proteins accessions identified per extract is indicated in Figure 2. All methods considered, number of identifications ranged from 48 (JBe2d4i1) to 93 (HAe2d2i2). On average for Jersey breed, there were 82.3 (±4.4), 55.5 (±4.2), and 69.1 (±4.2) protein accessions identified, respectively for extracts A, B, and C. On average for Holstein-Friesian breed, there were 81.6 (±4.3), 66.6 (±4.2), and 72.8 (±3.3) protein accessions identified, respectively for extracts A (urea), B (TCA/acetone), and C (methanol/chloroform). Unexpectedly, while extracts C (methanol/chloroform) produced the highest recovery rate and the highest concentrations relative to extracts A (urea) and B (TCA/acetone), they generated less unique accessions than extracts A (urea), albeit more than B (TCA/acetone).

**Figure 3:**
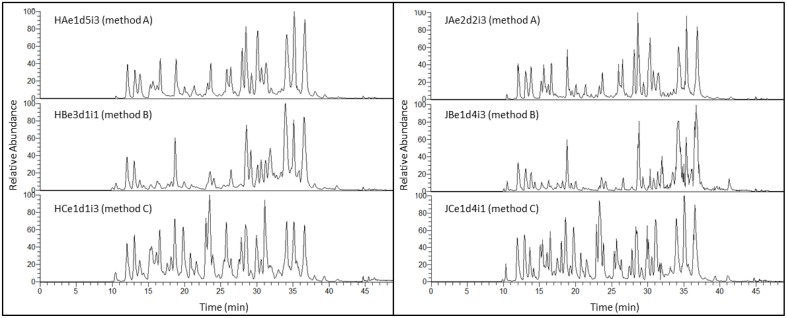
**Total Ion Chromatograms (TIC) of three tryptic digests illustrating the effect of extraction method for Holstein-Friesian (left panel) or Jersey (right panel) breed**. A TIC represents the summed intensity across the entire range of masses being detected at every point in the analysis. The duration of each nLC run is 50 min (x-axis), with tryptic peptides eluting from 10 to 42 min. Relative abundance (percent relative abundance with respect to the ion of highest abundance along the y-axis) of the most intense chromatographic peaks are comparable across methods. Most abundant peaks elute toward the end of the nLC run (27–38 min) for methods A (urea) and B (TCA/acetone), while they are evenly distributed along the whole elution pattern (11–38 min) for method C (methanol/chloroform). Subtle differences in peptide elution are visible between Holstein-Friesian (left panel) and Jersey (right panel) breeds. The nomenclature of each TIC exemplified here is explained in the Materials and Method Section and in Figure 1.

Figure 3 shows TICs of three tryptic digests from Holstein-Friesian and Jersey breeds illustrating the effect of the extraction methods. Peptides eluted from around 10 to 42 min. The three methods generated distinct TICs, with method C (methanol/chloroform) displaying peaks with higher resolution than methods A (urea) and B (TCA/acetone). The peaks eluting from 19.5 to 20.5 min, and 22.5 to 24 min, and which were the most intense in samples processed using method C (methanol/chloroform), yielded several peptides from α-S1-caseins. TICs are much more comparable across breeds than across methods because the elution patterns look similar, yet subtle differences can be seen in Figure 3 between the left and right panels, particularly with respect to the relative abundance of the chromatographic peaks. This is an indication that protein complexity varies between Holstein-Friesian and Jersey breeds, not only in a quantitative manner, as demonstrated with the protein concentrations, but also qualitatively. Indeed, different proteins will produce different tryptic peptides. This carries through to PCA plots as they were derived from chromatographic peaks, as illustrated below.

### Reproducibility

**Figure 4:**
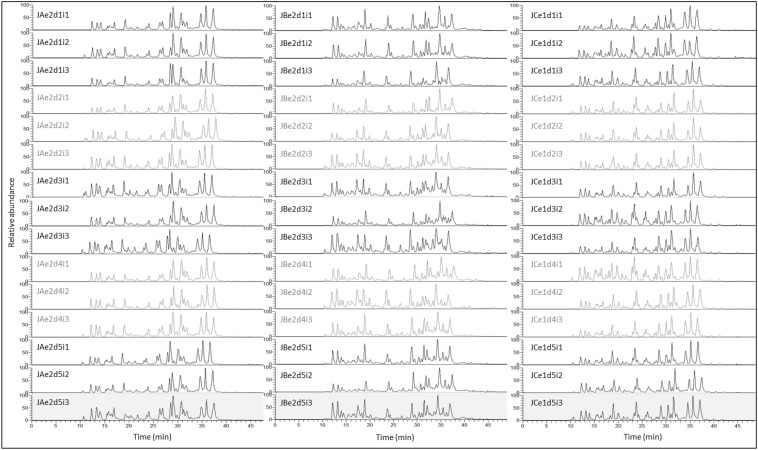
**TIC of 45 Jersey tryptic digests illustrating the reproducibility at digestion (5 replicates) and injection (3 replicates) levels, for methods A (urea) (15 replicates), B (TCA/acetone) (15 replicates), and C (methanol/chloroform) (15 replicates)**. TICs of each set of three randomized repeated injections are alternatively black or gray. The x-axis represents the duration of the nLC run in min, while the y-axis represents the relative abundance of the chromatographic peaks which corresponds to the percent relative abundance with respect to the ion of highest abundance. With the exception of the inconsistent peptides eluting very early (10–12 min) or very late (39–42 min) during the 50 min nLC run, TICs are very reproducible across technical replicates, within a particular method. The nomenclature of each TIC exemplified here is explained in the Materials and Method Section and in Figure 1.

Figure 4 illustrates a complete set of 15 replicates resulting from one extract for each method (5 individual digestions and three randomized repeated injections). Apart from the first and last peaks, TICs are very reproducible within a method, particularly within a set of 3 repeated injections (i1, i2, and i3).

**Figure 5:**
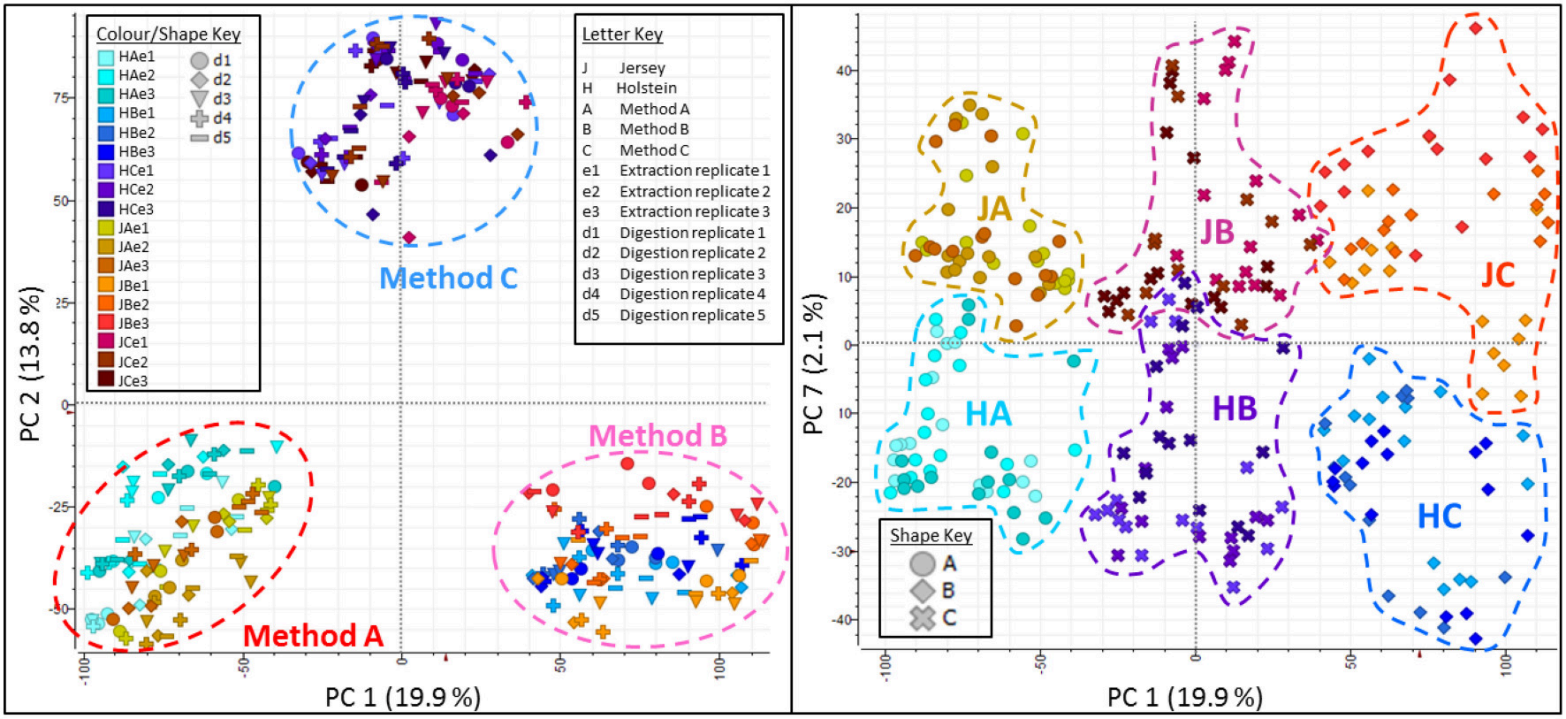
**Principal component analyses (PCA) plots along Principal Component (PC) 1 against PC2 (left panel), and PC1 against PC7 (right panel)**. Together PC1 (19.9%) and PC2 (13.8%) explain 33.7% of the total variance and clearly separate the three methods. Within each method, all replicates cluster together whether it be at the extraction, digestion or injection levels. Breed explain 2.1% of the variance along PC7. On the plot PC1 against PC7, methods and breed are well-separated.

**Figure 6:**
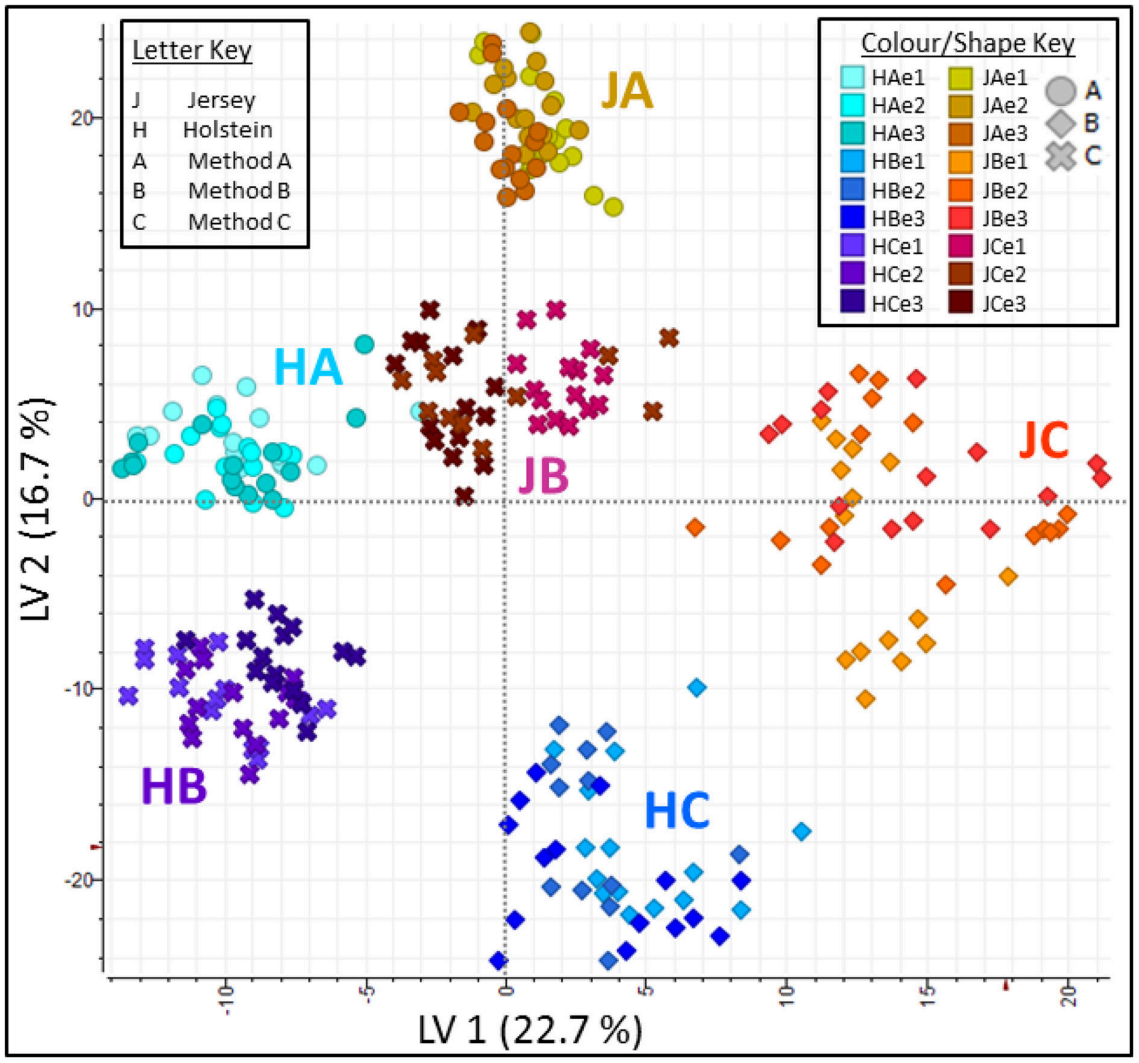
**Partial Least Square (PLS) analysis plots along Latent Variable (LV) 1 against LV2**. Together LV1 (22.7%) and LV2 (16.7%) explain 39.4% of the total variance, with a clear separation of breeds and methods, and displaying six tight clusters for JA, JB, JC, HA, HB, and HC.

Principal Component Analyses of the MS data highlighted the reproducibility of the individual extraction methods while showing there were clear differences between the different methods (Figure 5). Principal Component (PC) 1 explained 19.9% of variance and clearly separated method A (urea) from method B (TCA/acetone). Principal Component 2 explained 13.8% of variance and set method C (methanol/chloroform) well apart from the other two methods. Within each method, all replicates clustered together whether it be at the extraction, digestion or injection levels. Within methods, cow breeds did not cluster together; it was evident within method A (urea) where Holstein-Friesian and Jersey breeds bear two different shades of colors that seldom mix. Breed explained 2.1% of the variance along PC7. On the plot PC1 against PC7, methods and breed were clearly separated. The effect of both cow breed and extraction method on protein analyses was further explored by PLS using only peaks which successfully led to protein identifications during database search (Figure 6). Plots of Latent Variable (LV) 1 (22.7%) against LV2 (16.7%) discriminated between breeds and methods, displaying 6 tight clusters for JA, JB, JC, HA, HB, and HC.

### Protein identities

**Figure 7:**
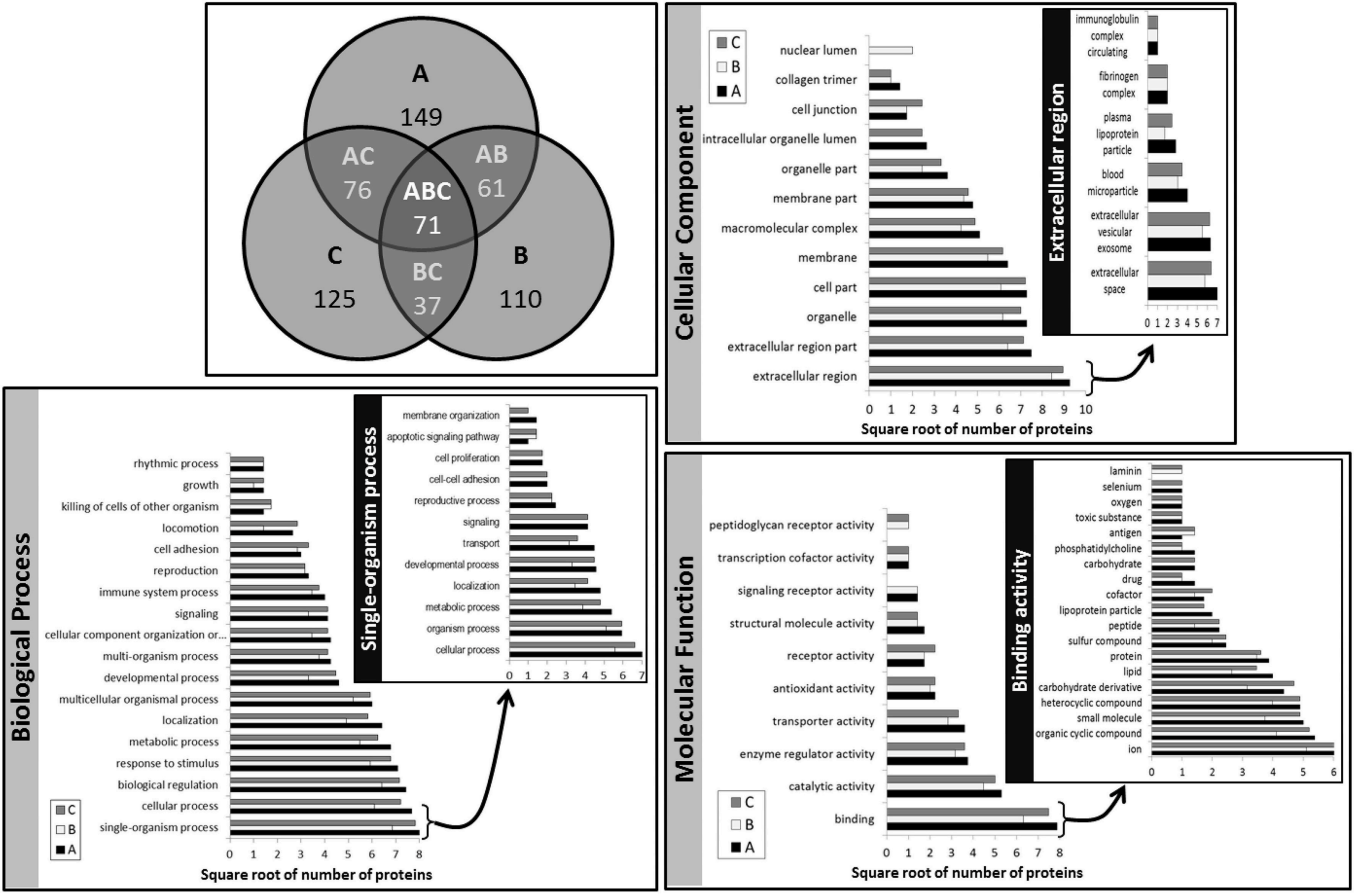
**Venn diagram of the number of unique protein accessions and Gene Ontology (GO) classification of known proteins per extraction method**. A, method A (urea); B, method B (TCA/acetone); C, method C (methanol/chloroform); AB, methods A and B combined; AC, methods A and C combined; BC, methods B and C combined; ABC, methods A, B, and C combined. On the histograms illustrating GO classifications, the x-axis represents the square root of the number of proteins belonging to each of the classes distributed along the y-axis. The insets illustrate the histograms of the sub-classes of the GO class containing the greatest number of proteins.

Table 1 lists all the unique protein accessions and reports in which method/breed they were identified. Accessions that were unique to a particular set of extracts or conversely shared among samples were summed and plotted as a Venn diagram (Figure 7). Numbers of unique accessions sorted as follows: 149, 110, and 125, respectively for extracts A (urea), B (TCA/acetone) and, C (methanol/chloroform). A total of 71 protein accessions were common to all methods. Methods A (urea) and C (methanol/chloroform) shared a large number of protein identities (76); 61 accessions were shared between extracts A (urea) and B (TCA/acetone); 37 accessions were shared between extracts B (TCA/acetone) and C (methanol/chloroform). Such representation highlighted the fact that as different as methods A (urea), B (TCA/acetone) and, C (methanol/chloroform). were from each other, they recovered the same types of proteins from skim milk samples. In total, 186 different protein accessions were identified across all methods. Identities common to all three sets of extracts include: caseins (α-S1, α-S2, β, and κ forms), lactoferrin, albumin, β-lactoglobulin, α-lactalbumin, complement C3, and butyrophilin. This was expected as these proteins are the most abundant in milk. Yet proteins present in low abundance in milk were also identified, such as enzymes and minor glycoproteins, as well as many immunoglobulins (Igs), antibodies, and antigens.

Gene Ontology classifications of known proteins are presented in Figure 7. All considered, classifications were very similar across methods, with method B (TCA/acetone) generally displaying the smallest number of proteins per category. As expected the most prominent protein category in the “Cellular Component” classification was the “extracellular region” as most milk proteins are secreted. The inset further details such components without revealing much difference across methods. Method B (TCA/acetone) had a unique “nuclear lumen” component due to ribonucleases, however it lacked the “intracellular organelle lumen.” Most “Molecular Functions” of identified proteins fell into the binding category, further detailed in Figure 7 inset. Method B (TCA/acetone) was lacking the “selenium binding” activity of selenium-binding protein 1. Method A (urea) was lacking the “laminin binding” function as it was depleted of dystroglycan. The peptidoglycan receptor activity was only found in methods B (TCA/acetone) and C (methanol/chloroform) and was associated to peptidoglycan recognition protein 1. The most prevalent Biological Process was “single-organism process,” detailed in the inset of Figure 7.

### Protein validation

**Figure 8:**
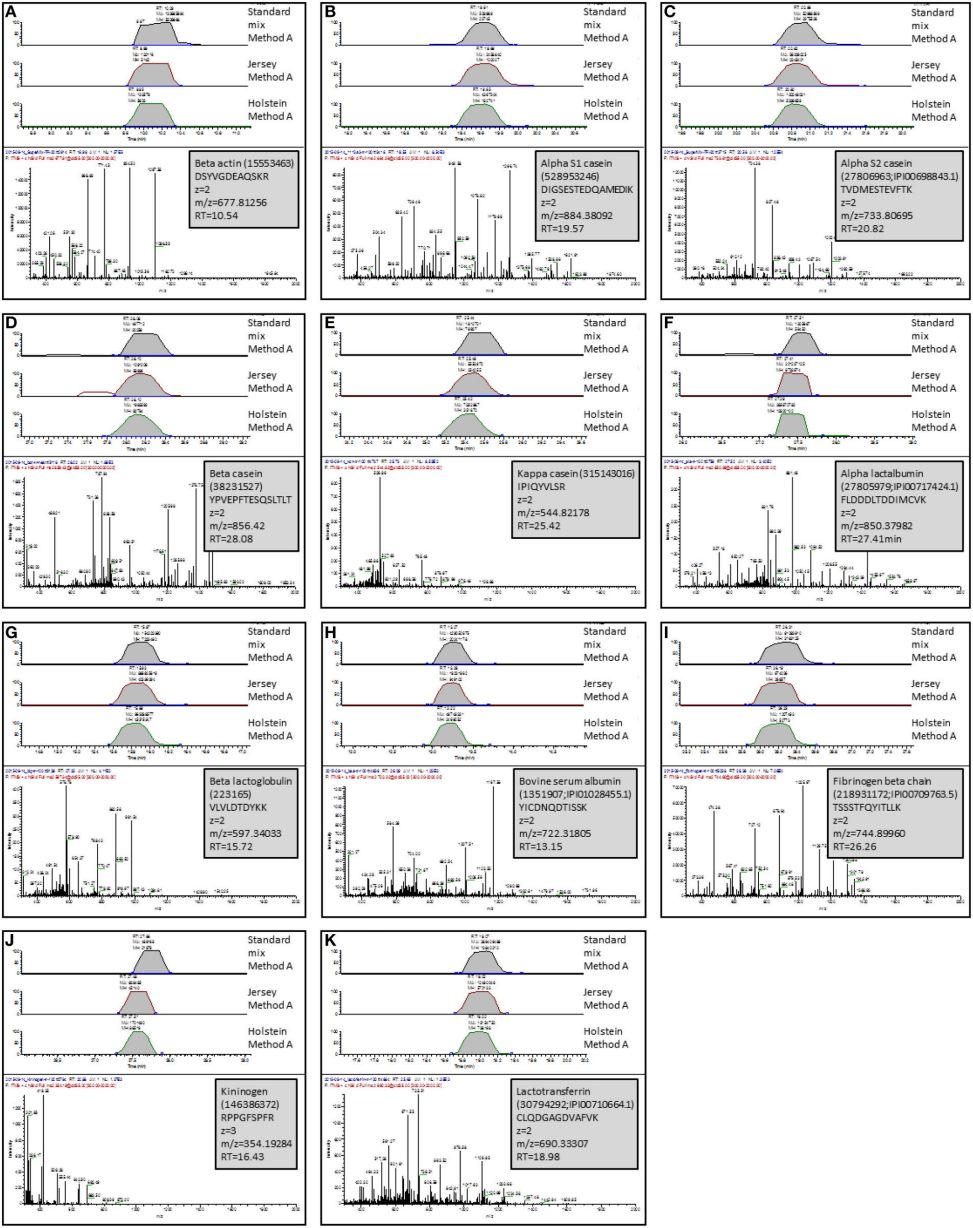
**Validation of protein identities using known protein standards**. One peptide per standard was selected and Extracted Ion Chromatograms (EICs) were produced and compared across the standard mixture, Jersey bulk milk, and Holstein bulk mik tryptic digests. Retention times (RT) are comparable across samples. The MS/MS spectrum of the selected peptide is displayed below the EICs. Insets indicate the proteins to which this peptide belongs, the AA sequence of the selected peptide, its m/z, charge state and RT. **(A)**, peptide from beta actin; **(B)**, peptide from alpha S1 casein; **(C)**, peptide from alpha S2 casein; **(D)**, peptide from beta casein; **(E)**, peptide from kappa casein; **(F)**, peptide from alpha lactalbumin; **(G)**, peptide from beta lactoglobulin; **(H)**, peptide from bovine serum albumin; **(I)**, peptide from fibrinogen; **(J)**, peptide from kininogen; **(K)**, peptide from lactotransferrin.

Using known protein standards, an independent experiment was designed on one hand to validate our shotgun nLC-MS/MS bottom-up approach and on the other hand, to confirm some of the proteins identified in our milk samples. To this end, actin, fibrinogen, lactoferrin, kininogen, α-casein, β-casein, κ-casein, α-lactalbumin, β-lactoglobulin, and BSA were purchased, and reconstituted in SB at the same concentration (10 mg/mL). These proteins were chosen as they displayed differences across methods and/or breeds. These standards were trypsin-digested individually and in combination, prior to analysis by shotgun nLC-MS/MS. Table 2 summarizes the identification results in the mixture of protein standards combined prior to trypsin digestion. Because their level of purity varied from 65 to 98%, our shotgun nLC-MS/MS approach identified proteins other than the known standards. The expected proteins were correctly identified with high scores (from 66 to 3415) and a mimimum of two Peptide Spectrum Matches (PSM, from 2 to 207) thereby validating our bottom-up identification method. For each of the know protein standard, a peptide was chosen, its Extracted Ion Chromatogram (EIC) was produced and compared across the standard mixture, Jersey and Holstein tryptic digests (Figure 8). The peptides were successfully found in all digests from the standard mixture and the milk sample; and they eluted at comparable retention times. This validates the protein identities from cow's milk samples.

**Table 2:** **List of protein accessions identified in the combined protein standards, along with their description, their score, coverage, the number of peptides identified per protein, the number of peptide spectrum matches (PSM), the size of the protein (AA and MW) and their theoretical isoelectric point (calc. pI)**.

**No**.	**Accession**	**Description**	**Score**	**Coverage (%)**	**# Peptides**	**# PSM**	**# AAs**	**MW [kDa]**	**calc. pI**
1	IPI00843089.3	11 kDa protein	124.2	29.0	2	2	107	11.1	7.99
2	**306440544**	**Actin**	**746.4**	**28.6**	**9**	**21**	**371**	**41.3**	**5.22**
3	**74267962**	**Albumin ALB protein [Bos taurus]**	**1905.6**	**51.7**	**29**	**60**	**607**	**69.2**	**6.25**
4	IPI00691212.1	Alpha-1-acid glycoprotein	84.3	15.4	3	3	202	23.2	5.87
5	**11036998**	**Alpha-lactalbumin [Physeter catodon]**	**94.0**	**12.0**	**1**	**2**	**92**	**10.4**	**4.59**
6	**27805979; IPI00717424.1**	**Alpha-lactalbumin precursor [Bos taurus]**	**858.8**	**31.0**	**18**	**51**	**142**	**16.2**	**5.14**
7	**528953238**	**Alpha-S1-casein isoform X3 [Bos taurus]**	**1009.1**	**40.8**	**8**	**37**	**206**	**23.6**	**5.19**
8	**528953244**	**Alpha-S1-casein isoform X6 [Bos taurus]**	**639.0**	**30.2**	**5**	**22**	**199**	**22.6**	**4.79**
9	**528953246**	**Alpha-S1-casein isoform X7 [Bos taurus]**	**1011.8**	**47.5**	**8**	**37**	**177**	**20.2**	**5.41**
10	**159793227**	**Alpha-S1-casein, partial [Bos taurus]**	**154.4**	**15.8**	**2**	**3**	**95**	**10.9**	**4.56**
11	**27806963; IPI00698843.1**	**Alpha-S2-casein precursor [Bos taurus]**	**650.6**	**38.7**	**19**	**64**	**222**	**26.0**	**8.43**
12	513137422	Antibody Blv1h12	221.5	23.2	3	5	216	22.5	6.11
13	114052298; IPI00688815.2	Apolipoprotein A-II precursor [Bos taurus]	73.4	16.0	2	2	100	11.2	8.10
14	47564119; IPI00689034.1	APOLIPOPROTEIN C-III precursor [Bos taurus]	108.3	16.7	2	4	96	10.7	5.11
15	41386683; IPI00686769.1	Beta-2-microglobulin precursor [Bos taurus]	148.9	18.6	6	9	118	13.7	8.00
16	**223165**	**Beta-lactoglobulin**	**736.4**	**48.2**	**9**	**26**	**162**	**18.3**	**4.92**
17	**87196497; IPI00699698.1**	**Beta-lactoglobulin precursor [Bos taurus]**	**1112.4**	**38.8**	**17**	**79**	**178**	**19.9**	**5.02**
18	**593730995**	**Beta-lactoglobulin-like [Physeter catodon]**	**65.6**	**9.4**	**2**	**2**	**180**	**20.3**	**5.94**
19	**1351907; IPI01028455.1**	**Bovine serum albumin**	**3415.0**	**69.4**	**78**	**207**	**607**	**69.2**	**6.18**
20	124056491; IPI00713505.2	Complement C3	179.6	4.5	10	10	1661	187.1	6.84
21	**6980814**	**Fibrinogen**	**908.6**	**32.6**	**11**	**21**	**390**	**42.7**	**7.97**
22	**75812954; IPI00691819.1**	**Fibrinogen alpha chain precursor [Bos taurus]**	**1601.7**	**41.1**	**40**	**80**	**615**	**67.0**	**7.17**
23	**1346006**	**Fibrinogen beta chain**	**741.2**	**37.6**	**13**	**20**	**468**	**53.3**	**8.19**
24	**488508027**	**Fibrinogen beta chain isoform 2 [Dasypus novemcinctus]**	**126.4**	**8.8**	**3**	**3**	**433**	**50.0**	**7.39**
25	**229156**	**Fibrinopeptide B**	**152.4**	**71.4**	**1**	**4**	**21**	**2.4**	**4.44**
26	528940100; IPI01028178.1	Fibronectin isoform X10 [Bos taurus]	292.9	5.0	14	14	2268	249.0	5.63
27	113912055; IPI00695142.3	Glycoprotein 2 (zymogen granule membrane) [Bos taurus]	81.7	2.8	2	2	534	59.2	4.82
28	343197008	Immunoglobulin lambda light chain IGLC2c [Bos taurus]	160.6	42.5	3	4	106	11.4	8.59
29	310893435	Immunoglobulin light chain [Bos taurus]	118.9	30.7	2	2	101	10.4	6.48
30	**1705608**	**Kappa-casein**	**472.7**	**22.4**	**4**	**25**	**192**	**21.5**	**5.81**
31	**284626**	**Kappa-casein–bovine**	**171.6**	**24.5**	**2**	**13**	**53**	**6.0**	**5.11**
32	**315143016**	**Kappa-casein [Bos indicus]**	**2183.5**	**36.8**	**5**	**90**	**144**	**15.9**	**6.77**
33	**284027124**	**Kappa-casein [Ovis vignei]**	**464.0**	**15.4**	**4**	**32**	**162**	**18.0**	**6.15**
34	**229416**	**Kappa-casein para kappaA**	**2020.9**	**46.7**	**4**	**79**	**105**	**12.3**	**8.78**
35	**162807**	**Kappa-casein precursor, partial [Bos taurus]**	**589.3**	**42.4**	**4**	**28**	**99**	**10.6**	**5.24**
36	**146386372**	**Kininogen [Oryctolagus cuniculus]**	**104.6**	**3.6**	**1**	**3**	**302**	**33.3**	**6.52**
37	27806851; IPI00716157.1	Lactoperoxidase precursor [Bos taurus]	58.8	1.5	2	2	712	80.6	8.54
38	**586476652**	**Lactotransferrin [Chrysochloris asiatica]**	**233.3**	**4.1**	**2**	**6**	**708**	**78.0**	**8.51**
39	**30794292; IPI00710664.1**	**Lactotransferrin precursor [Bos taurus]**	**2949.1**	**57.2**	**88**	**179**	**708**	**78.0**	**8.32**
40	528912092; IPI00685784.3	Neutrophil gelatinase-associated lipocalin isoform X3 [Bos taurus]	168.8	16.0	4	6	200	23.0	9.17
41	528961411; IPI00866916.1	Plasminogen isoform X3 [Bos taurus]	103.2	4.6	3	4	724	81.5	7.96
42	3914346; IPI00696714.1	Polymeric immunoglobulin receptor	130.9	5.9	5	5	757	82.4	7.27
43	95006989; IPI00760446.1	Ribonuclease 4 precursor [Bos taurus]	122.2	28.6	6	6	147	16.9	8.85
44	118150406; IPI00824879.1	Secretoglobin family 1D member precursor [Bos taurus]	83.1	21.6	4	4	102	11.3	8.73
45	2501351; IPI00690534.1	Serotransferrin	117.9	7.7	6	6	704	77.7	7.08
46	554537890	Serotransferrin [Myotis brandtii]	70.8	4.1	2	2	713	77.9	7.05
47	24119203; IPI00714405.3	Tropomyosin alpha-3 chain isoform 2 [Homo sapiens]	51.4	4.0	2	2	248	29.0	4.78
48	**999627**	**Trypsin**	**77.5**	**22.0**	**2**	**3**	**82**	**8.8**	**7.30**
49	IPI00843209.1	Uncharacterized protein	228.2	18.7	7	7	443	50.2	5.72
50	IPI00712994.3	Uncharacterized protein	532.4	29.2	6	23	161	18.3	5.19
51	556760750	Uncharacterized protein LOC102338350 [Pantholops hodgsonii]	60.0	4.1	1	4	245	28.1	9.70

*As the purity level of each standard varied, expected known proteins are highlighted in bold*.

## Discussion

The intended aim of this study was to establish a procedure to extract proteins from cow milk with minimum steps prior to protein digestion and shotgun nLC-MS/MS analyses, which yielded high protein concentration and was reproducible. To this end, three extraction methods were performed on skim milk samples from Jersey and Holstein-Friesian cows, resulting in three protein extracts. These methods were chosen because they were based on very different chemistries yet were simple enough to be performed in a high-throughput fashion as discussed below. As far as we know, these methods have not previously been applied to bottom-up proteomics of samples of cow milk.

Method A (urea) merely consisted of a 50% dilution of skim milk samples with an urea-based solubilisation buffer. Urea is a common chaotrope used in the solublization and denaturation of proteins; by unfolding proteins urea uncovers buried disulphide bonds accessible to reduction and modification. The solubilisation buffer contained DTT to reduce protein disulfide bridges over the 30 min incubation at 30°C, while incubation temperature was purposefully kept well below 35°C so as to inhibit the carbamylation of proteins which may occur in presence of urea. Reduced disulphide bridges were further stabilized using the alkylating reagent IAA. The solubilisation buffer also contained the anionic detergent SDS which disaggregates casein micelles as well as NaCl which influences their physico-chemical stability. The solubilisation buffer was buffered at pH 8.0 using Tris-HCl to improve the stability of denatured/reduced milk proteins. Similar procedures have been employed in which full cream milk samples were skimmed and diluted in a different urea-based buffers prior to 2-DE; such buffers contained 8 M urea, 40 mM Tris, 2 or 4% CHAPS, 50 or 65 mM DTT, and 0.2 or 2% ampholytes as they improve protein focusing during IEF (Boehmer et al., 2008; Jensen et al., 2012a; Yang et al., 2013). We cannot compare the efficiency of extraction of our method A to that of the reports afore-mentioned as the downstream analytical method employed here is different. Furthermore, these reports did not aim at improving protein extraction. Yet, most of the proteins identified by Boehmer et al. (2008) and Jensen et al. (2012a) were also identified in extracts A. In the present study, of the three methods, method A (urea) was by far the simplest and the quickest necessitating only one dilution step, therefore introducing the least variation due to sample handling. However, because method A (urea) does not include a centrifugation step and produced a fully soluble extract devoid of precipitate, it should not remove non-protein compounds thus potentially interfering with subsequent steps.

Method B (TCA/acetone) resulted from a simple acetone precipitation procedure under cold, reducing and acidic pH conditions, commonly used in proteomics notably on plant and fungal tissues (Vincent et al., 2005, 2007, 2009, 2012a,b; Vincent and Solomon, 2011) and known as a TCA/acetone precipitation. Acetone reduces the dielectric constant of water and displaces the water molecules surrounding proteins during precipitation, thereby leading to strong hydrophobic interactions between proteins followed by aggregation. The addition of TCA lowers the pH and promotes hydrophobic aggregation by not only disrupting the solvation layers of the proteins but also furthering protein denaturation thereby exposing more hydrophobic surface to the solvent. Dithiothreitol reduces disulfide bonds. Solvent precipitation must be performed at subzero temperatures in order to minimize protein degradation. By removing solvent-soluble compounds such as polar metabolites, method B (TCA/acetone) should result in protein-enriched extracts. While we could not find a publication reporting the use of TCA/acetone to extract cow milk proteins, an acetone-precipitation method was applied to study the inflammation of bovine mammary glands in full cream milk samples as part of the iTRAQ extraction and labeling procedure, resulting in the quantitation and identification of up to 169 proteins (Danielsen et al., 2010). It is possible that more proteins could have been recovered by using a different extraction method, however, iTRAQ manufacturer imposes such acetone precipitation. In another instance, proteins were removed by acetone precipitation prior to Carbograph-4 cartridge elution in order to enrich aflatoxin M1 levels in milk samples (Cavaliere et al., 2006). Again more proteins could have been targeted by using a different removal method, yet, acetone is a solvent compatible with graphitized carbon black cartridge. Therefore, whether used as an enrichment method or a depletion method, acetone successfully precipitated proteins in both studies cited above. Most proteins identified by Danielsen et al. (2010) were also identified in extracts B. Whilst straightforward, method B (TCA/acetone) involved a precipitation step and two washing steps, interspersed with centrifugation steps which made this protocol more labor-intensive, time-consuming and subject to more variation than method A (urea).

Method C (methanol/chloroform) (Taylor and Savage, 2006) arose from modifications brought to the Bligh-Dyer chloroform/methanol partition procedure (Bligh and Dyer, 1959). This protocol was initially designed to rapidly extract lipids from wet cod fish muscles, which contain 80% water and 1% lipids. It operates on the principle that the water contained in the sample becomes miscible with a chloroform/methanol solution (1:2 by volume). Further addition of one volume of chloroform and one volume of water creates a biphasic partition where the lipids solubilise in the chloroform layer whereas the non-lipid compounds go into the methanolic layer. The original Bligh-Dyer procedure was subsequently modified by substituting water with a 8% NaCl solution (Taylor and Savage, 2006), thus blocking the binding of some acidic lipids to denatured lipids. This method was successfully applied to recover fatty acids from mussel tissues (Taylor and Savage, 2006). As methanol is a solvent used in proteomics to precipitate proteins, notably following phenol extraction (Vincent et al., 2006, 2009), and because most proteins are insoluble in chloroform, partition protocols such as method C (methanol/chloroform) produce an interphase between the lower chloroform layer and the upper methanol layer that contains milk proteins and is free of most lipids and metabolites, therefore purifying proteins from non-protein compounds. Method C (methanol/chloroform) is routinely used in our lab to extract fatty acids in the chloroform phase from full cream milk samples prior to GC-MS analyses (Ezernieks et al., unpublished data) while polar metabolites are recovered from the methanol phase to undergo LC/MS analysis (Elkins et al., unpublished data). To our knowledge, method C (methanol/chloroform) has never been applied to recover proteins from milk samples. However, comparable methods have been employed as exemplified hereafter. Touati et al. (1992) demonstrated that, in chloroform/methanol solution (1:1 by volume), the solubility of caseins and β-lactoglobulins varied in a pH dependent fashion as it affected the neutralization of milk protein polar functions. More recently, following chloroform/methanol extraction, the milk fat globule membrane fraction displaying the highest anti-rotavirus activity was shown to be highly non-polar and devoid of proteins (Fuller et al., 2013). Method C (methanol/chloroform) was as time-consuming as method B (TCA/acetone), yet more intricate as it required the recovery of the protein interphase. In our hands, the use of a swing bucket rotor during the centrifugation step instead of a fixed-angle rotor (data not shown) increased interphase stability so much so that the paper-thin interphase could be gently pushed aside while the upper and lower liquid phases were tipped out. Method C (methanol/chloroform) involved various steps possibly impacting reproducibility. It also used greater extraction solution volumes than methods A (urea) and B (TCA/acetone), which necessitated larger tubes to the detriment of throughput during the centrifugation step. Placed into a systems biology context, method C (methanol/chloroform) is highly advantageous as it allows the recovery of polar, non-polar metabolites and proteins in one step. This would allow proteomics and metabolomics studies to be conducted on the same sample.

If we were to compare the three methods based on their duration and cost, again method A (urea) would outperform the other two methods as it takes much less time, effort and money to complete the protein extraction from milk samples. The time required for method A (urea) is 2.5 h whereas the time required for method B (TCA/acetone) or C (methanol/chloroform) involves 5 h extraction and overnight incubation. Methods B (TCA/acetone) and C (methanol/chloroform) are as time-consuming. Method C (methanol/chloroform) is more labor-intensive and requires more skills, particularly when recovering the protein interphase. Furthermore, as opposed to method A, methods B and C include centrifugation steps which not only limit the throughput of the protocols but also add time. The cost, based on chemicals, associated with method A (urea) is minimum ($0.09 per sample) as opposed to method B (TCA/acetone) which costs fifty times more than method A ($4.45 per sample) and method C (methanol/chloroform) which costs 12 times more than method A ($1.05 per sample). Method B (TCA/acetone) is four times more expensive than method C (methanol/chloroform).

In its principle, method A (urea) did not seek to enrich protein content like method B (TCA/acetone) or to purify proteins like method C (methanol/chloroform). Method C (methanol/chloroform) outperformed the other methods when SDS-PAGE patterns, protein concentration and protein recovery rates were considered regardless of the breed, thus confirming that cow milk proteins were more specifically extracted by a tri-phasic partition procedure. Following extraction, the same amount of proteins underwent trypsin digestion per extract, thereby eliminating concentration variations across methods and breed. Digestion and subsequent clean-up steps using SPE and UF of the tryptic peptides were performed uniformly in a rigorous manner for all samples. Differing greatly in their chemistry, each method produced distinct chromatograms during the nLC-MS/MS analyses. Method A (urea) yielded the greatest number of accessions relative to methods B (TCA/acetone) and C (methanol/chloroform), suggesting that extracts A (urea) were compatible with the various steps post-extraction. While method C (methanol/chloroform) was superior to the other methods in most respects as demonstrated by protein assays, SDS-PAGE patterns consistently exhibiting the most prominent proteins, α-caseins, and TICs, it did generate fewer accessions than Method A (urea). We could hypothesize that the preponderance of α-caseins masked the presence of other proteins, and were preferentially targeted during trypsin digestion to the detriment of minor proteins. Indeed the most prominent chromatographic peaks of samples processed using method C (methanol/chloroform) eluted tryptic peptides from α-S1-caseins, the most abundant of all milk proteins. Method B (TCA/acetone) consistently yielded the least optimum results showing little compatibility with downstream analyses; consequently we do not recommend its application for milk samples. All three methods were highly reproducible as demonstrated by the TICs traces and PCA plots, with overall samples originating from method A (urea) generated tighter clusters. This probably arose from the fact that method A (urea) had less steps than methods B (TCA/acetone) and C (methanol/chloroform), therefore less subject to experimental variation. Functional classification did not highlight categories unique to a method because as different as the three methods were, they recovered similar proteins, extracts B (TCA/acetone) generally having less of them. Based on these findings, we reject our hypothesis that all methods are similar in terms of their major attributes, and we recommend either method A (urea) or method C (methanol/chloroform) to extract proteins from cow milk samples in gel-free bottom-up approach.

As expected in our study, the most abundant milk proteins were identified across all three methods: caseins (α-S1-, α-S2-, ß-, and κ-forms), ß-lactoglobulin, α-lactalbumin, lactoferrin, and lactoperoxidase. Apart from the major milk proteins, many immunoglobulins (Igs) were also identified. These immunoglobulins belonged to the main classes IgG1, IgG2, IgA, and IgM. Immunoglobulins protect both cow udders and offspring from microbial infections and their abundances fluctuate with cow species, breed, age, stage of lactation, and health status (reviewed in Marnila and Korhonen, 2011). Other proteins involved in the bovine immune defense system identified in the present study included ß_2_-microglobulin and osteopontin (Wynn et al., 2011 for review). A number of enzymes were found in our protein samples, including the well-studied lipoprotein lipase (LPL). Lipoprotein lipase is a glycoprotein involved in fatty acid synthesis and triggering rancidity in milk and its derivative products (Deeth, 2011). Another enzyme was sulfhydryl oxidase (SOx) which catalyses the disulphide bond formation essential to the three-dimensional structure of proteins (reviewed in Farkye and Bansal, 2011). Another enzyme was identified in all three methods and both cow breeds, xanthine dehydrogenase/oxidase (XOR) which commonly occurs in the milk fat globule membrane (MFGM). Xanthine dehydrogenase/oxidase enzymatic role makes it a source of reactive oxygen and nitrogen species; XOR also displays antimicrobial activities (reviewed in Harrison, 2011). Another enzyme identified in this work is β-1,4-galactosyltransferase 1 (Gal-T1) involved in the synthesis of complex carbohydrates decorating glycoproteins and glycolipids and whose affinity for its substrates is regulated by α-lactalbumin, which is also a glycoprotein (Brew, 2011). Beta-1,4-galactosyltransferase 1 was identified in all extracts and breeds along with various glycoproteins (butyrophilin subfamily 1 member A1, lactadherin, lactotransferrin, lactoperoxidase, mucins 1 and 15, Igs, α-1-acid glycoprotein, α-1B-glycoprotein, α-2-HS-glycoprotein, pancreatic secretory granule membrane major glycoprotein GP2, platelet glycoprotein 4, Zn-α-2-glycoprotein) as well as glycosylation-dependent cell adhesion molecule 1, dystroglycan, and peptidoglycan recognition protein 1. The prominence of glycoproteins in cow milk was reflected in our results, yet surprisingly little is known about their biological funtions; the carbohydrate moieties play an essential communication role in numerous cellular processes (O'Riordan et al., 2014).

Several studies have compared top-down analyses of intact milk protein variants from Holstein-Friesian and Jersey breeds (Jensen et al., 2012a,b; Poulsen et al., 2013; Gustavsson et al., 2014). These studies focussed on the most abundant proteins such as caseins, α-lactalbumin and β-lactoglobulins. As far as we know, there are no publications using a bottom-up proteomics strategy to compare milk proteins from Holstein-Friesian and Jersey cows. In this study, bulk milk samples representing whole Holstein-Friesian and Jersey herds were analyzed using many replicates. Our results highlighted proteins that were more prominent in one breed compared to the other (Table 1). For instance, a fatty acid-binding protein was 30% more abundant in Holstein-Friesian milk than Jersey milk. This protein facilitates the transfer of fatty acids between extra- and intracellular membranes. This is may be relevant as Holstein-Friesian and Jersey milk fat content and composition differ, with Jersey milk fat containing higher concentrations of saturated fatty acids, especially of fatty acids with short and medium carbon chains (Arnould and Soyeurt, 2009). Alpha-A 2-HS-glycoprotein, also known as fetuin-A which forms soluble complexes with calcium and phosphate, was 28% more prevalent in Holstein-Friesian milk than in Jersey milk. This could be related to the fact that Holstein-Friesian milk contains less total calcium than Jersey milk (Jensen et al., 2012b). Conversely, actin 1, a globular multi-functional protein that forms microfilaments found in all eukaryotic cells, was occurring 26% more in Jersey milk than in Holstein-Friesian milk. The significance of this finding is unclear at this stage. Two proteins involved in angiogenesis and cellular protein synthesis, lactadherin and angiogenin-1, occurred more in Holstein-Friesian milk than Jersey. The prevalence of fibrinogen (alpha and beta subunits), a glycoprotein complex involved in blood clot formation, in Holstein-Friesian milk relative to Jersey remains to be further investigated. A vitamin D-binding protein and cathelicidin-4, whose levels accumulate with those of vitamin D during an infection (Liu et al., 2006), were more prominent in Holstein-Friesian milk than Jersey milk. Two serpins (serpin A3-1 and α-1-antiproteinase, also called α-1 anti-trypsin or AAT1) prevailed in Holstein-Friesian milk. These serine protease inhibitors activity protects tissues from damage caused by proteolytic enzymes; AAT1 is the most abundant serpin in human (Hunt and Tuder, 2012). The anti-microbial proteins peptidoglycan recognition protein 1 and histatherin, also known as histatin, occurred more in Holstein-Friesian than Jersey milk. Several proteins involved in the immune system underpinned breed difference: Antibodies prevailed in both milks with IgG2 isotype more prevalent in Holstein-Friesian milk. CD5L scavenger receptor protein prevailed in Jersey milk. Combined together, these findings suggest that milk varies in protein species composition and that dairy cattle breeds may have evolved different milk qualities. Many of the differences relate to immune proteins and responses.

These results remain preliminary findings as the proteomic analysis was optimized using bulk milk samples which represent a whole herd. Further studies are underway to investigate the profile of these specific proteins in individual Holstein-Friesian and Jersey cows. They will shed light on genetic differences.

## Conclusions

In this study, three protein extraction methods performed on bulk milk samples from Jersey and Holstein-Friesian cows were compared using protein assay, SDS-PAGE, and nLC-MS/MS analyses. All major milk proteins such as caseins were extracted along with less abundant proteins such as whey proteins (β-lactoglobulin, α-lactalbumin, lactotransferrin), as well as minor proteins such as glycoproteins, and enzymes. Method A (urea), a simple dilution of milk into an urea-based buffer, yielded the greatest number of unique protein accessions. Method B (TCA/acetone) was not as efficient as methods A (urea) and C (methanol/chloroform). Method C (methanol/chloroform) yielded the highest protein concentration, recovery rates, as well as best SDS-PAGE patterns. Such a tri-phasic partition procedure would be highly desirable for experiments assessing the inter-relationships between metabolites and protein regulation in milk such as in systems biology projects. However, for a proteomics-centric approach, method A (urea) offers advantages in low costs, simplicity, protein coverage and throughput and would be the preferred method for this type of study.

## Funding

This work was funded by Department of Economic Development, Jobs, Transport, and Resources.

### Conflict of interest statement

The authors declare that the research was conducted in the absence of any commercial or financial relationships that could be construed as a potential conflict of interest.

## Abbreviations

A: urea method
AAT1: alpha-1 anti-trypsin
B: TCA/acetone method
C: methanol/chloroform method
d1 to d5: digestion replicate 1 to digestion replicate 5
e1 to e3: extraction replicate 1 to extraction replicate 3
Gal-T1, beta-1: 4-galactosyltransferase 1
H: Holstein-Friesian cows
i1 to i3: injection replicate 1 to injection replicate 3
Ig: immunoglobulin
J: Jersey cows
LPL: lipoprotein lipase
MFGM: milk fat globule membrane
SB: Solubilisation Buffer
SOx: sulfhydryl oxidase
XOR: xanthine dehydrogenase/oxidase.
